# Cryo-EM structure of the Seneca Valley virus A-particle and related structural states

**DOI:** 10.1128/jvi.00744-25

**Published:** 2025-08-20

**Authors:** Rosheny Kumaran, Nadishka Jayawardena, Kuan-Lin Chen, Alice-Roza Eruera, James Hodgkinson-Bean, Laura N. Burga, Matthias Wolf, Mihnea Bostina

**Affiliations:** 1 Department of Microbiology and Immunology, University of Otago 626306, Dunedin, Otago, New Zealand; 2 Molecular Cryo-Electron Microscopy Unit, Okinawa Institute of Science and Technology Graduate University 56874, Kunigami District, Okinawa Prefecture, Japan; 3 Theoretical Sciences Visiting Program, Okinawa Institute of Science and Technology Graduate University (OIST), Onna-son, Okinawa, Japan; University of Michigan Medical School, Ann Arbor, Michigan, USA

**Keywords:** picornaviruses, capsid, cryo-electron microscopy, Seneca Valley virus genome uncoating

## Abstract

Picornavirus cell entry requires a series of capsid protein conformational changes leading to genome uncoating. For enteroviruses, receptor binding triggers the transition from a full (F) capsid to an altered (A) particle before releasing its genome and finally converting it into an empty (E) particle. In contrast, non-enteroviruses, such as Aphthovirus, Cardiovirus, or Seneca Valley virus, release their genomes by dissociating the capsid into pentamers. While the existence of a transient A-particle for non-enteroviruses was previously speculated, it has never been directly observed using structural methods. Seneca Valley virus (SVV) is an oncolytic picornavirus that selectively targets cancer cells by recognizing Tumor endothelial marker 8 (TEM8) as the host receptor. SVV disassembles into pentamers at acidic pH, suggesting that the acidic environment of the endosome could cause capsid disassembly. We used cryo-electron microscopy to investigate SVV under acidic conditions and in complex with TEM8 at physiological pH, identifying multiple uncoating intermediates. These include an altered-particle, an empty-rotated particle (E^R^), and a series of open particles expelling the coiled genome. The A-particle is expanded, displays reduced interactions between capsid proteins, a reorganized genome, and has a poorly resolved VP1 N-terminus, VP2 N-terminus, and VP4. The E^R^ particle has rotated pentamers, reduced contacts within the particle, lacks the genome, VP1 and VP2 N-termini, and VP4. Our work provides an understanding of transient SVV structural states and supports the existence of an intermediate SVV A-particle. These findings could help optimize SVV for oncolytic therapy.

**IMPORTANCE:**

Seneca Valley virus (SVV) is a non-enterovirus picornavirus with specific tumor tropism mediated by the receptor Tumor endothelial marker 8, also known as Anthrax toxin receptor 1. Using cryo-electron microscopy, it was possible to identify multiple structural states of SVV. We demonstrate that SVV capsids transition from full particles to altered (A) particles and then to empty-rotated (E^R^) particles, with receptor binding and acidic pH driving these conformational changes, respectively. This study also identifies open particles with expelled genomes. Comparisons between A- and E^R^-particles reveal that peptide segments of VP1, VP2, and VP4 could potentially play a role in genome delivery. Future work can explore the formation of these structural states *in vivo*.

## INTRODUCTION

The *Picornaviridae* family is the largest viral family, with around 68 genera infecting a wide range of hosts (1–3). To date, many picornavirus capsid structures have been solved using either cryo-electron microscopy (cryo-EM) or X-ray crystallography (4), with enteroviruses being the most extensively studied members. Substantial effort has been devoted to understanding the variety of structural states that can be adopted by the virus. However, structural states and transition mechanisms of non-enterovirus picornaviruses remain poorly understood. Picornavirus capsids serve a dual purpose: they encase and protect the viral genome from external damage while being adaptable enough to release their genome under optimal conditions into the host cell (3, 5–19). The structural states and genome release mechanism of enteroviruses, such as Poliovirus, Enterovirus, Rhinovirus, Coxsackievirus, and Echovirus, have been extensively studied. The 160S full (F) capsids of the enteroviruses convert into a 135S altered (A) particle at either physiological pH, low pH, upon receptor binding, after chemical treatment, or after heating. The A-particles are characterized by an expanded capsid, collapsed hydrophobic pockets in VP1, the absence of pocket factors, genome reorganization, externalized N-terminus of VP1, released VP4, and widening of the gap of an axis of the capsid (5–10, 12–17, 20–26). Enterovirus E-particles lack a genome but share similar structural characteristics with the A-particle (7, 9–12, 15, 16, 20, 22–24, 27–30).

Enteroviruses may employ different processes for genome release; it could either be released by endosomal rupture (31) or translocated through the endosomal membrane (32, 33). Tomography studies have shown that genome containing enteroviruses such as Rhinovirus, Echovirus, and Enterovirus exit the endosome via a fundamental cellular process of endosomal rupture to initiate an infection (31). Although the limited resolution did not allow definitive identification of A-particles in the endosome environment, a small number of E-particles were observed. These E-particles likely released their genome prior to endosomal rupture, lowering the probability of successful infection due to potential RNase degradation (31). Alternatively, past research has shown that enteroviruses release their genome from within the endosome, and regions of VP1 and VP4 facilitate this process while protecting it from RNase degradation (5, 6, 17, 19, 21, 32, 34–39). The widening of a gap around the twofold axis in the A-particle of Poliovirus was shown to serve as a conduit for genome release, leading to the formation of an 80S empty (E-) particle (8, 28). Interestingly, in some enteroviruses, such as Echovirus 18, genome release occurs through the expulsion of one or several pentamers, accompanied by the release of the coiled genome (22).

Past studies on non-enterovirus picornaviruses have identified structural states that differ from enteroviruses. Cardioviruses form an A-particle followed by an E-particle, which later disassembles into pentamers (40). Aichi virus-1 forms expanded E-particles without forming an A-particle (41). Aphthoviruses, such as the foot-and-mouth disease virus (FMDV) and Equine rhinitis A virus (ERAV), form different structural states; FMDV disassembles into pentamers that can reassemble into an “inverted” capsid, while ERAV forms an expanded E-particle with rotated pentamers (42, 43). This demonstrates the diverse nature in which these viruses have evolved the ability to form various structural states.

Seneca Valley virus (SVV) is a non-enterovirus and the single member of the Seneca Valley virus genus that was classified into three clades: clade I comprises the oncolytic strain SVV001, while clades II and III are associated with swine vesicular disease, a serious agricultural pathogen that is difficult to control and one of the primary causes of acute piglet death worldwide (44). SVV has a typical picornavirus capsid with four capsid proteins; VP1, VP2, and VP3 are characterized by a single jelly roll fold, where eight beta-strands form a barrel-like structure that is distributed at the exterior of the virus, while VP4 is located on the interior of the capsid (45). The structural architecture consists of four capsid proteins forming a protomer, 5 protomers forming a pentamer, and 12 pentamers assembling into a pseudo T=3 capsid, approximately ~32 nm in diameter (45). SVV specifically identifies and kills cancer cells by recognizing Tumor endothelial marker 8 (TEM8), also known as Anthrax toxin receptor 1 (ANTXR1), a cell surface receptor highly expressed in cancer cells. Structural data confirm that TEM8 binds to SVV at the BC loop and Loop II of VP1, the Puff of VP2, and the Knob of VP3 (46), preserving the capsid conformation present in the F-particle (45). SVV also forms naturally occurring empty capsids called procapsids, structurally similar to the F-particle (47) that may serve to deter neutralizing antibodies and store pentamers for later virion assembly and further RNA packaging (47).

Previous studies have shown that SVV disassembles into pentamers at acidic pH, suggesting the endosomal environment triggers structural changes (47, 48). However, receptor binding at pH 6 is required to trigger structural changes, indicating a role for receptor interaction in the genome uncoating process (48). Although enteroviruses are known to detach from the receptor at endosomal pH (31), it is not known whether SVV can still engage with its receptor during/after capsid disassembly, potentially stabilizing a structural intermediate at pH 5. This could provide structural insight into the potential structural states formed by SVV. Additionally, understanding SVV’s behavior under acidic conditions may inform its potential use as an oncolytic virus in low-pH environments such as tumors in the gastrointestinal tract (49). In addition, investigating the structure of SVV with TEM8 under physiological conditions would allow us to better understand the structural states that could potentially form *in vivo*.

We resolved the structure of SVV under acidic conditions and in the presence of TEM8 under physiological conditions using cryo-EM and single-particle analysis. Both conditions triggered the formation of distinct SVV structural states, which involve capsid dissociation and genome exposure. We reconstructed the cryo-EM maps of the altered particle, the empty-rotated (E^R^) particle, and a series of open (O-) particles releasing the coiled genome. The discovery of these states provides insights into the structural changes that the SVV capsid can undergo.

## RESULTS

We used cryo-EM and single-particle analysis to investigate the structural behavior of Seneca Valley virus capsids under different conditions. We observed several structural states at acidic pH [C] and in complex with TEM8 under physiological conditions [P]. All data sets were handled independently throughout processing, reconstruction, and modeling. In both cases, we observed various SVV structural states that provide valuable information on the dynamic nature of the SVV capsid.

### Receptor binding triggers partial exposure of the viral genome

**Fig 1:**
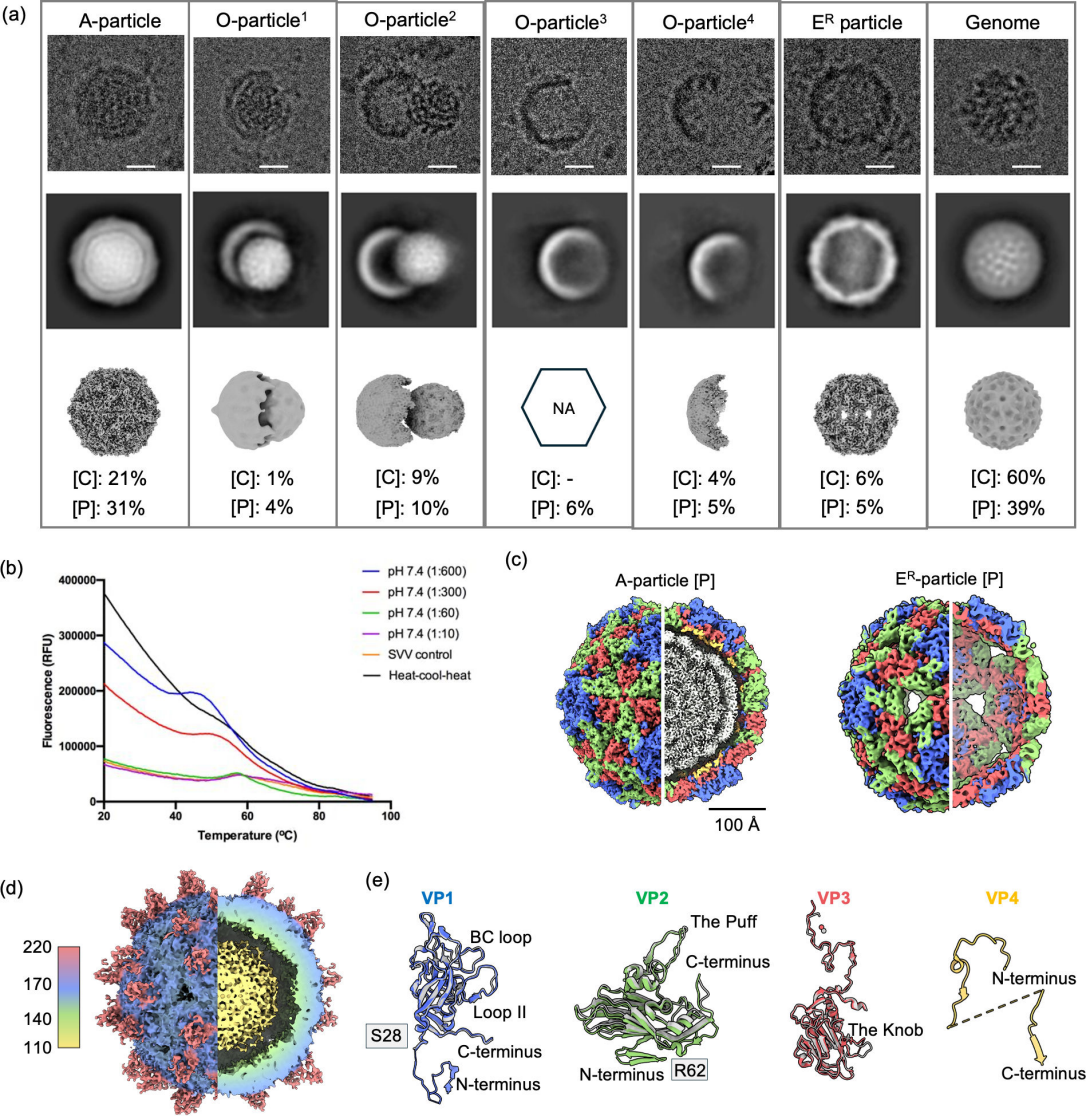
Characterization of SVV particles under acidic conditions and physiological conditions with TEM8. (**a**) SVV particles were visualized under both physiological [P] and acidic [C] conditions. The top, middle, and bottom rows show representative micrographs, 2D class averages, and 3D reconstructions, respectively. Reconstructions were generated using their respective symmetry as detailed in Materials and Methods (Fig. S2 and S3). Percentages indicate the distribution of particles in each structural state. Scale bars represent 100 Å. NA represents structures that were not solved. (**b**) Particle stability thermal release assay was performed at pH 7.4 to assess the thermal stability of SVV in the presence of varying virus: TEM8 receptor ratios. The SVV control (yellow) reflects SVV only, while the 1:600 virus: receptor condition underwent a heat–cool–heat cycle with incubation at 37°C followed by cooling to 4°C. (**c**) Cutaway views of the A-particle[P] and E^R^-particle[P] are shown at a threshold of 0.6. (**d**) Lowering the threshold to 0.11 reveals additional density corresponding to the TEM8 receptor on the E^R^-particle surface. (e) Structural comparisons between the A-particle[P] (colored) and E^R^-particle[P] (gray) monomers show that the E^R^-particle lacks the N-terminus of VP1, the N-terminus of VP2, and the whole of VP4. This was also observed in the E^R^-particle[C].

It was previously shown that TEM8 binds to the SVV capsid with a dissociation constant of (0.088 ± 0.010 *K*_*d*_ [S^−1^]) (48). Interestingly, we found that RNA exposure at 20°C was virus-to-receptor ratio dependent (Fig. 1b). At a 1:60 virus-to-receptor ratio, fluorescence intensity was similar to that of the SVV control, suggesting that this ratio may not trigger RNA exposure. However, at higher virus-to-receptor ratios—1:300 and 1:600—fluorescence intensity increased by approximately 100,000-fold and over 200,000-fold, respectively, indicating potential genome exposure. Both published structures of the SVV-TEM8 complex were obtained after incubation at 37°C, followed by cooling to 4°C or incubation at 4°C alone (46, 48). Therefore, any conformational changes exposing the RNA genome in the SVV-TEM8 complex were not accessible at the time of imaging in any of these studies. Overall, the data suggest that receptor binding at pH 7 and 37°C induces structural changes that promote genome exposure.

### Exposure of SVV to acidic conditions or in complex with TEM8 under physiological conditions generates various capsid structural states

We used cryo-EM to image SVV particles after incubation at pH 5 and 37°C with purified TEM8 to explore the structural behavior of SVV. We also imaged SVV complexed with TEM8 under physiological conditions (pH 7 and 37°C). In both cases, electron micrographs revealed a series of distinct structural states (Fig. 1a), with capsids appearing expanded, empty, or open.

Although the number of particles was limited, we selected enough particles to obtain 3D reconstructions. After careful classification, we obtained icosahedral reconstructions revealing two types of capsids: an expanded altered particle containing a genome and an empty particle with “rotated” pentamers, which we will refer to as the E^R^-particle (Fig. 1c). These structural states were observed under both conditions, though following slightly different distributions. For both physiological and acidic conditions, the A-particles were resolved to 3.4 Å, while for the E^R^-particles, the resolutions were 6.4 and 4.3 Å, respectively (Table S1; Fig. S1).

Enteroviruses imaged under physiological conditions, acidic conditions, or bound to their receptor transition to A- and E-particles while retaining a small population of F-particles (7, 10, 15, 16). In the case of SVV, we did not observe F-particles under any condition. The population of E^R^-particles was just ~5% for both acidic conditions and complexed with TEM8 under physiological conditions. Interestingly, in addition to the A- and E^R^-particles, we observed numerous instances of open particles with their genome expelled (Fig. 1a). We could not reconstruct any O-particles to high resolution, likely due to their inherent flexibility, heterogeneity, and the low number of particles in the micrographs (Fig. S2 and S3). However, SVV capsids missing a single pentamer were still rigid enough for a meaningful reconstruction (Fig. S4). Localized refinements of the SVV O-particles show that genome release is accompanied by the loss of a different number of pentamers, ranging from one up to half of the capsid (Fig. S2 and S3). While the preferential particle orientation of pentamers in ice prevented a high-resolution reconstruction, we refer to our previous study (47) for details on pentamer structure.

### Structure of the capsid proteins in the A- and E^R^-particles

In the F-particle, no density was observed for VP2 residues 1–11 or VP4 residues 1–13 and 39–62. This is also the case for the newly identified particles. For the A-particle[P], additional disordered regions without interpretable density include VP1 (G13, S20, and S28), VP2 (D12), and VP3 (E61-S66), while for the A-particle[C], these include VP3 (P60-D67). In the E^R^-particle[P], these include VP1 (S1-G27), VP2 (D12-D61), and VP4. Similarly, in the E^R^-particle[C], these include VP1 (S1-S28), VP3 (P61-S66), and VP4 (Fig. 1e). Disordered regions lacked interpretable density, making it challenging to confidently build a model in these areas.

All four capsid proteins were identified in both A-particle maps, but some regions were only visible at lower thresholds (below 0.80), suggesting they were partially ordered (Fig. 3). On the capsid exterior, the A-particle[P] had the following partially ordered regions: the VP1 HI loop (S229), VP1 BC loop (A65), VP1 Loop II (G97), and VP2 residues V59 and D67, while the A-particle[C] exhibited similar behavior showing partially ordered regions: VP1 HI loop (T230) and VP1 Loop II (P95-G97) (Fig. 3). The Knob region in both A-particles could not be modeled, which aligns with previous observations in SVV-TEM8 and SVV particles at pH 6, pH 7, and pH 8 (45, 47, 48). At the interior, the A-particle[P] displayed partially ordered regions in the VP1 N-terminus (S1-S28), VP2 N-terminus (R13-G20), VP3 FG loop (G160-N162), and VP4 N-terminus (S38, G63-K72). For the A-particle[C], the disordered regions were on the VP1 N-terminus (E11-S28) and VP4 residues G63 to N66 (Fig. 3).

The atomic models for both A-particles are similar (Fig. S6), with all-atom RMSDs of VP1, VP2, VP3, and VP4 being 0.563, 0.465, 0.522, and 0.980 Å, respectively. The VP1 protein conformations have high structural similarity (Fig. S6a). VP2 residues L85-P86, S151-E158, F175-S177, K249-D255, and A278-G279, and VP3 residues F79-D80 and L204-C208 display minimal shifts (Fig. S6b and c). Changes in the structure of individual capsid proteins in the A-particle under acidic conditions relative to the F-particle (Protein Data Bank [PDB] ID: 3CJI) are minimal. In VP2, residues L85, N122, L152-E158, W173-S177, Y181-Y182, R183-W187, E250-A252, and A209-R210 shift, however, not by more than 2.0 Å (Fig. S7b), which could be explained by the resolution of the map. There is minimal difference between the two VP3s (Fig. S7c).

### The A-particle is expanded relative to the F-particle

The A-particle is visibly larger than the native F-particle (PDB ID: 3CJI) as measured between the center of the atomic model of opposing pentamers. The A-particles obtained in both conditions are expanded by ~5% in diameter compared to the F-particle (Table S2). This expansion results in reduced capsid protein contacts compared to the F-particle. Inter-pentamer contacts are reduced by 34% and 22% under acidic and physiological conditions, respectively. Protomer and pentamer contacts are reduced by ~6% and ~14%, respectively (Fig. S5). The models were built in their respective maps and show minor differences in side-chain densities. Some side chains were removed during modeling, potentially resulting in the observed differences between the A-particles.

### The E^R^-particle is larger than the A-particle

**Fig 2:**
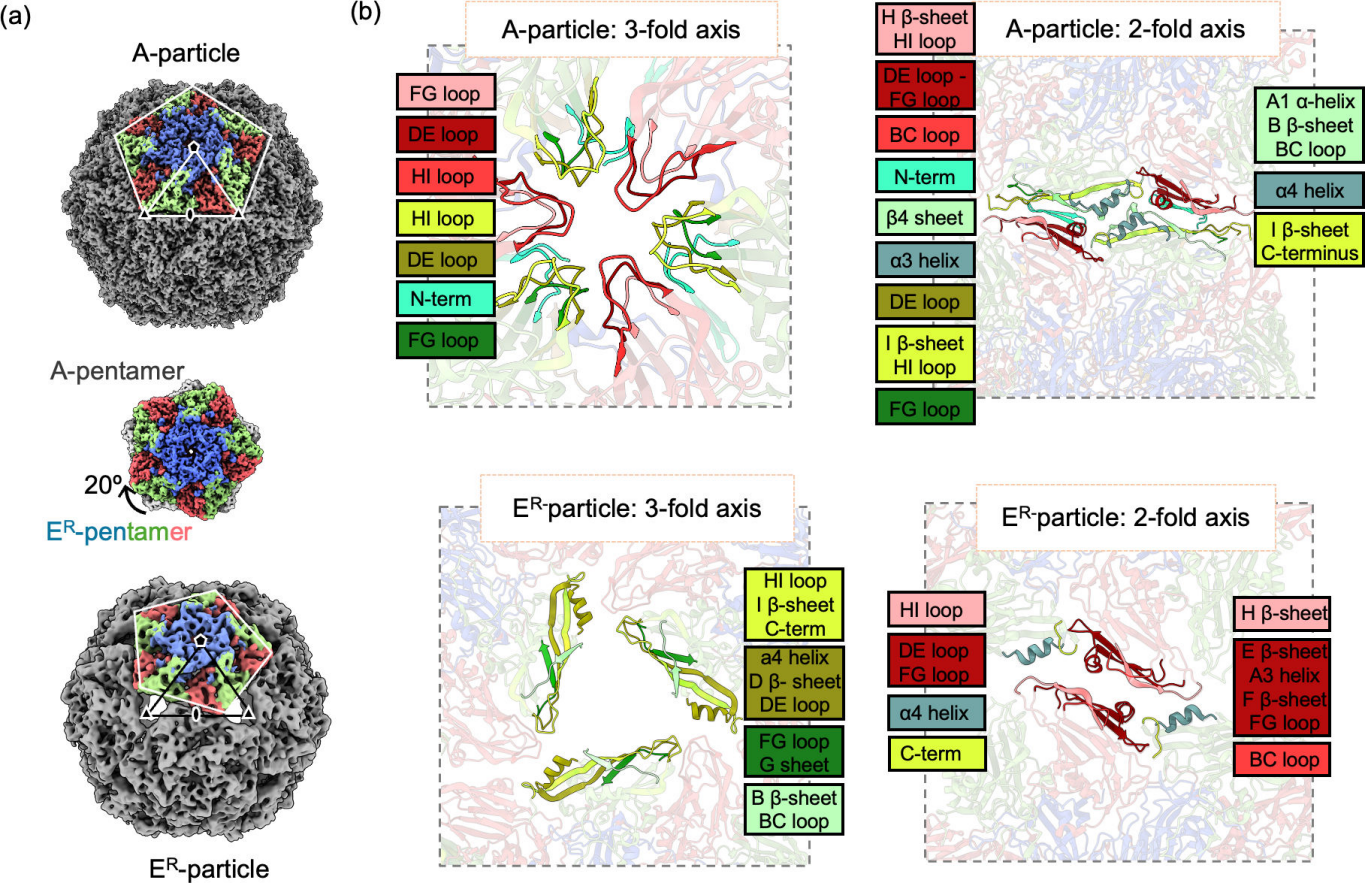
Structural changes in A- and E^R^-particles. (**a**) Cryo-EM reconstructions of A-particle[P] and E^R^-particle[P] at a threshold of 0.6, with five-, three-, and twofold symmetry axes marked by pentagons, triangles, and ovals, respectively. In the E^R^-particle, each pentamer is rotated approximately 20° clockwise relative to its position in the A-particle. This shift is illustrated in the middle panel, where an E^R^-particle pentamer (colored) is superimposed on an A-particle pentamer (gray). A similar rotational shift is observed under acidic conditions. (**b**) Detailed views of the three- and twofold axes show how pentamer rotation changes the inter-pentamer contacts. In the E^R^-particle, rotation reduces VP2–VP3 interactions and introduces new VP2–VP3 and VP3–VP3 contacts between pentamers at the twofold axis. VP2 (green) and VP3 (red) subunits are shown with corresponding labels to highlight changes in positioning. Labels on the left indicate VP2 and VP3 pairs facing each other at the axis, while labels on the right show the same monomers facing each other in the new arrangement.

Although the E^R^-particle from both conditions remains icosahedral, there is a distinct rearrangement of pentamers. Each pentamer has rotated approximately 20° clockwise compared to its position in the F- and A-particles (Fig. 2a). As a result, the E^R^-particle is 8% larger than the A-particle and 14% larger than the F-particle, representing a ~44% increase in volume compared to the native state (Table S2). A similar conformation was previously reported for SVV by exposing the receptor-decorated capsids at pH 6 (48).

### Genome organization in the A-particle

In the F-particle, the genome is partially ordered as a dodecahedral cage (47). Partially ordered genomes were previously reported in other picornaviruses and were proposed to play a role in genome uncoating (30, 50). Our maps show that this structure is maintained in the A-particle but without the inner capsid contacts. Once released, the genome loses its dodecahedral organization, expanding by 1%–4% in diameter while retaining a spherical appearance (Table S2; Fig. S8). This behavior can be attributed to the existence of ordered RNA that is not spontaneously untangled. Interestingly, while investigating the SVV capsid behavior, we occasionally observed extended strings of RNA alternating with unfolded regions in negative stain micrographs. Remarkably, all these regions had an elongated profile and were distributed at regular intervals (Fig. S8b). These observations suggest that RNA plays an active role during genome uncoating. It is tempting to speculate that maintaining a compact arrangement is necessary during the transition of the RNA through the cellular membrane before its disentanglement in the cytoplasm, where replication takes place.

### Inter-pentamer interactions in the A-particle and E^R^-particle

The rotation of the pentamers in the E^R^-particle leads to dramatic rearrangements. In the A-particle, inter-pentameric contacts at the threefold axis occur mainly between VP2 and VP3: the N-terminus, HI, DE, and FG loops of VP2 in one pentamer face the DE, FG, and HI loops of VP3 in the other pentamer (Fig. 2b; Fig. S9). This rotation creates a triangular hole at the threefold axis (Fig. 2b; Fig. S9).

In the A-particle, the inter-pentameric contacts at the twofold axes are primarily between VP2 and VP3. The N-terminus, β4 sheet, α3 helix, DE loop, I β-sheet, HI loop, and FG loop of VP2 in one pentamer face the BC loop, DE loop to FG loop region, HI β-sheet, and the HI loop of VP3 in the other pentamer (Fig. 2b; Fig. S9). The E^R^-particle has novel VP2-VP3 and VP3-VP3 contacts at the twofold axis. The α4 helix and C-terminus of VP2 face the DE loop, FG loop, and HI loop of VP3 in the other pentamer. The BC loop, E β-sheet, α3 helix, F β-sheet, and FG loop of VP3 from two pentamers face each other.

### A- and E^R^-particles have different charge and hydrophobicity profiles

The disordered N-terminus of VP1 and VP2 and the total absence of VP4 in the E^R^-particle lead to a different landscape on the capsid interior (Fig. 1). The disordered VP1 and VP2 regions are strongly negatively charged near the threefold axis in the A-particle (Fig. S10). The absence of these regions in the E^R^-particle makes the area slightly positively charged. The N-termini of VP1 and VP2, along with the entirety of VP4, comprise both hydrophobic and hydrophilic residues. The absence of these regions in the E^R^-particle exposes more hydrophobic regions on the interior of the capsid.

### Receptor density at varying conditions

**Fig 3:**
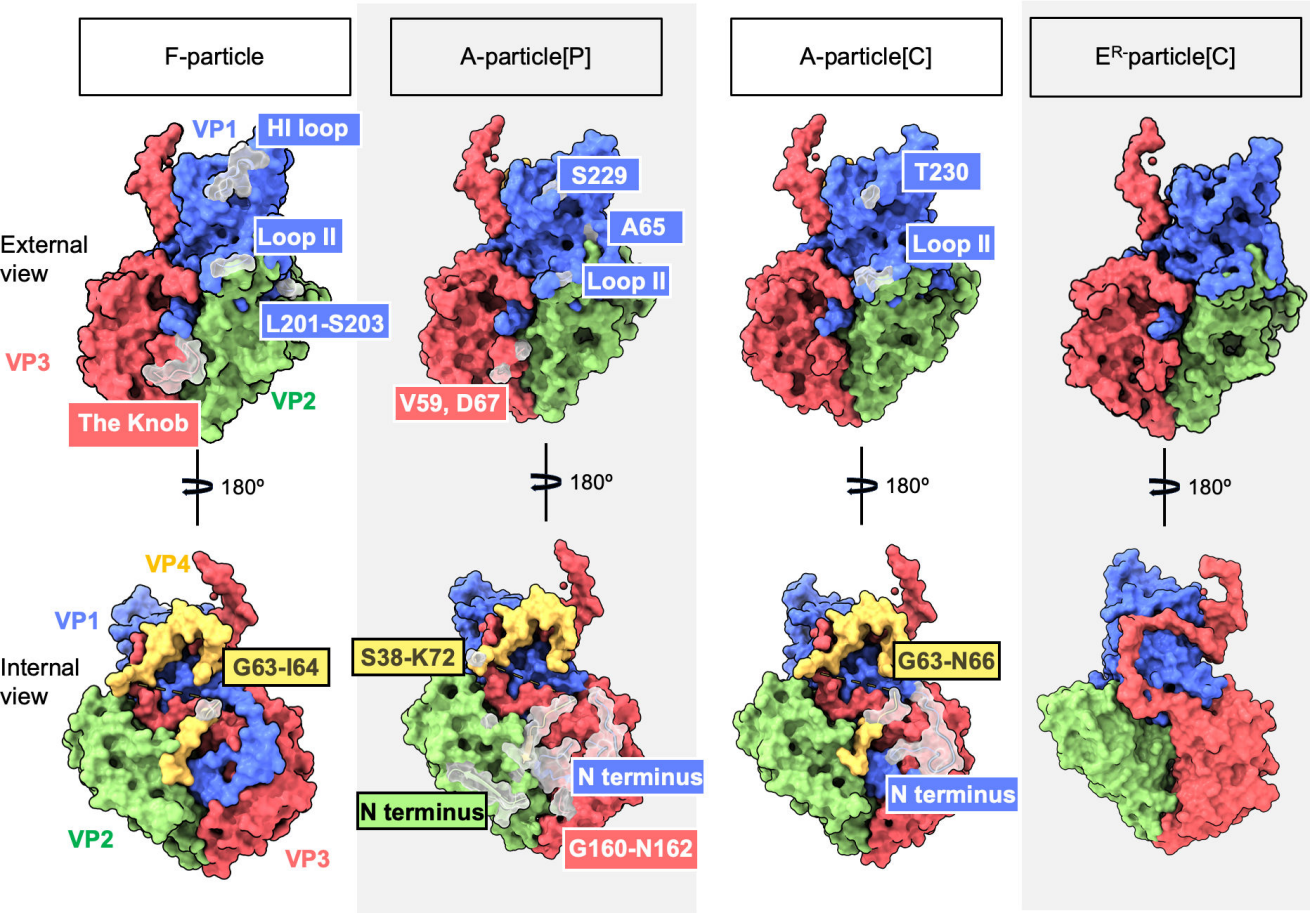
Structural comparison of the F-, A-, and E^R^-particles. The protomer subunits are colored: VP1 (blue), VP2 (green), VP3 (red), and VP4 (yellow). Protomers are shown with both external and internal views. Some regions were only visible at lower thresholds (F-particle below 0.0107 and A-particle below 0.80), suggesting they were partially ordered. The transparent surfaces represent the partially ordered regions. The F-particle structure is based on previously published data (EMD-7110, PDB: 3CJI), while A- and E^R^-particle models represent reconstructions under physiological [P] and acidic [C] conditions. In the F-particle, poorly resolved regions include the VP1 HI loop (E226-A231), Loop II (**S96-G99**), GH loop (L201-S203), VP3 Knob (**P60-D67**), and a segment of VP4 (**G63-I64**). The partially ordered regions on the exterior of the A-particles are VP1 Loop II (**P95-G97**), the HI loop (S229-T230), the BC loop (**A65**), and VP2 (**V59, D67**). On the interior of the A-particle, the regions are the VP1 N-terminus, VP2 N-terminus, VP3 FG loop (G160-N162), and regions of VP4.

Density corresponding to TEM8 was not visible on the SVV A- and E^R^-particles under acidic conditions, suggesting that at low pH, the virus potentially loses the affinity for the receptor or binds transiently. This is similar to the behavior demonstrated by other enteroviruses, where the receptor detaches from the capsid under acidic conditions (10, 31). Interestingly, under physiological conditions, no receptor density is observed on the A-particle[P]. However, density corresponding to TEM8 becomes visible on the E^R^-particle when the map is contoured to a low threshold of 0.11 (Fig. 1d) (46). In our previous structure of the SVV-TEM8 complex at 4°C, we have shown that TEM8 interacts with specific regions of SVV: the BC loop and Loop II (VP1), the Puff (VP2), and the Knob (VP3) (46). Our present results show that at least two out of four TEM8 binding sites are partially ordered in the F- and A-particles (Fig. 3). These flexible regions could also contribute to receptor interactions’ adaptability and potential reversibility (51). Our particle stability thermal release (PaSTRY) assay shows that a virus receptor ratio of 1:300 and above initiates the transition to the A-particle and further RNA exposure. Previously published structures of the SVV-TEM8 complex were obtained by incubating a 1:5 virus receptor ratio for 3 hours (46) and overnight (48), while our preparation was obtained at a 1:600 virus receptor ratio incubated for 90 minutes. This suggests that low virus receptor ratios may be insufficient for immediate binding to initiate capsid state transition at neutral pH (Fig. 1b). The receptor density on the E^R^-particle[P], although visible only at higher contour levels, supports the idea of functionally significant yet transient receptor interaction during capsid transition. The fact that TEM8 density becomes visible on the E^R^-particle under physiological conditions potentially suggests that the pentamers or the receptor may adopt a more favorable conformation, enabling a more rigid receptor attachment.

## DISCUSSION

We used cryo-electron microscopy to investigate the behavior of Seneca Valley virus capsids in highly acidic (pH 5) and neutral (pH 7) environments at 37°C in the presence of TEM8. Under both conditions, we detected distinct particle populations, including the altered particle and empty-rotated particle, as well as numerous open particle intermediates with different numbers of pentamers still associated.

Our findings reveal both shared and unique structural features between the SVV A-particle and those formed by enteroviruses, including those of Poliovirus, Coxsackievirus, Enterovirus, Echovirus, Human Rhinovirus, and Rhinovirus (5–10, 12–17, 20–24). In polioviruses, the A-particle is expanded in size and externalizes the VP1 N-terminus and VP4 as part of genome release (7). The SVV A-particle had poorly resolved density for the VP1 and VP2 N-termini and regions of VP4, suggesting flexibility and partial disorder. However, there was no visible density showing an externalized VP1 N-terminus in the SVV A particle. In Poliovirus, the major capsid proteins move away from each other in the A-particle, and the VP4, VP1, and VP2 N-termini detach from each other and become disordered (7, 23). The reduced capsid contacts within the SVV A-particle may aid in the flexibility of the VP1 N-terminus, VP2 N-terminus, and VP4. We also identified the presence of numerous O-particles and the globular appearance of the externalized genome. The presence of O-particles and a substantial population of coiled genomes (from both conditions) could suggest that SVV releases its genome first by expanding to the A-particle and then expelling pentamers (Fig. 1). This implies that the SVV A-particle is a transient capsid state that can transition into an O-particle. The genome expulsion phenomenon is like the uncoating mechanism previously reported for Echovirus 18 and Kashmir bee virus, belonging to the *Dicistroviridae* (22, 52). These findings suggest that viruses can undergo diverse structural strategies for genome release.

The SVV E^R^-particle displays structural characteristics that set it apart from the E-particle formed by enteroviruses. In enteroviruses such as Poliovirus, Coxsackievirus, Enterovirus, Echovirus, Human Rhinovirus, and Rhinovirus, the 80S E-particle is structurally similar to the A-particle, except that it lacks genomic RNA (7, 9–12, 15, 16, 20, 22–24, 27–30, 53). The SVV E^R^-particle also lacks its genome, but the capsid architecture differs from the E-particles formed by enteroviruses due to its distinct rotation of pentamers. The rotated pentamers also establish a new set of inter-pentamer contacts within the SVV capsid. Overall, the SVV E^R^-particle is defined by rotated pentamers, an expanded capsid, reduced inter-pentamer contacts, lacks the genome, the VP1 N-terminus, VP2 N-terminus, and the whole of VP4. A similar SVV E^R^-particle was identified at pH 6 in the presence of TEM8 (48), which lacked 43 residues at the N-terminus of VP2, whereas our E^R^-particle at pH 5 lacked 62, indicating the incremental impact of acidification on particle stability. The enterovirus A- and E-particles display the same expansion relative to the F-particle (7, 20). In contrast, the SVV E^R^-particle is 8% larger in diameter than the A-particle.

The observation of O-particles with very few E^R^-particles implies that the E^R^-particle could arise from pentamer reassociation after genome expulsion. Non-enteroviruses such as ERAV and FMDV from the *Aphthovirus* genus release their genome by dissociating into pentamers (42, 54). The disassembled FMDV capsid has been observed to reassemble into an inside-out capsid (42). The rearranged FMDV capsid has different antigenic properties, but its functional importance has not been explored (42). A similar E^R^-particle for ERAV was solved by cryo-EM at low resolution, showing a volume 42% larger than the F-particle and was proposed to be an uncoating intermediate (43). However, it has been speculated that the ERAV E^R^-particle is a result of pentamer reassociation following genome release, similar to the mechanism in FMDV (42). Our results support this scenario and suggest that our SVV E^R^-particle may represent a reassociation phenomenon of dissociated pentamers from genome release, as shown for enteroviruses. The functional importance of the SVV E^R^-particle is currently unexplored.

Notably, several regions of the capsid proteins that are partially ordered in the A-particle are absent in the E^R^-particle. The N-termini of VP1 and VP2, as well as the VP4, contain patches of hydrophobic residues that could associate with the lipid bilayer to anchor the capsid in the proximity of the membrane (Fig. S10). In enteroviruses such as Poliovirus and Rhinovirus, the N-terminus of VP1 and VP4 interact with lipid membrane to facilitate this process. The N-terminus of VP1 is externalized and anchors the capsid to the membrane (5, 6, 17, 21, 32, 35, 36). Meanwhile, VP4 is essential in forming a multimeric pore in the membrane, through which RNA is hypothesized to pass (37–39). Interestingly, for poliovirus, it was reported that long umbilical connectors at the quasi threefold axis potentially formed by the N-termini of VP1 or VP4 can facilitate RNA transfer from within the endosome (32). This mechanism not only enables genome delivery but was also found to protect the RNA from RNase degradation, suggesting it as a possible strategy for safe genome release (32, 33). Structural data from previously published studies of Poliovirus (6, 7, 23, 55, 56), Coxsackievirus A6 (25, 26), Enterovirus A71 (12, 53), SVV (45, 47), Cardiovirus (40), and FMDV (42, 57) were combined. For most viruses, the A- and E^R^-particles have no density for the N-terminus of VP1 and for VP4 in the E-particle. Future work into the role of the disordered regions in SVV could provide valuable insight into its role in capsid state transitions and potential genome release. The precise mechanism by which the SVV genome reaches the cytoplasm remains unknown; it could either be released by endosomal rupture (31) or translocated through the endosomal membrane (24, 32, 33).

Previously, we have shown that the VP1 N-terminus adopts an ordered conformation in the SVV F-particle when in contact with the genome but becomes poorly resolved in procapsids lacking the genome (47). In the SVV A-particle, its genome is reorganized and lacks defined capsid-RNA interactions with the inner capsid surface, likely contributing to the flexibility of the VP1 N-terminus, which is consequently poorly resolved. This reorganization may facilitate genome release, potentially through the formation of the O-particles. A similar genome distribution is observed in the human Cardiovirus Saffold F- and A-particles (40). Conversely, the EV71 and Rhinovirus 14 A-particle had its genome interacting with the capsid near the twofold axis for genome release (10, 12).

Our PaSTRy assay results show that TEM8 receptor binding to SVV at pH 7 induces structural changes that promote genome accessibility. The observed increase in initial fluorescence at 20°C with higher virus-to-receptor ratios potentially indicates a more pronounced expanded A-particle. We note that our structure obtained at pH 5 displays no TEM8 density despite being incubated with a high virus-to-receptor ratio. Past work on Rhinovirus and other enteroviruses has shown that acidic pH causes the receptor to detach (10, 31). Our findings also indicate that acidic conditions may ablate receptor binding, potentially due to pH-induced conformational changes in the receptor or receptor-binding region. The saturation of the particle with receptor molecules and a highly acidic environment may destabilize the capsid beyond a native state, affecting the relevance of the observed conformations to natural infection. However, our structures from pH 7 were captured at 37°C, below the critical temperature at which TEM8-saturated SVV falls apart (Fig. 1b). Under physiological conditions, no receptor density is observed on the A-particle[P]. The receptor density on the E^R^-particle[P], although visible only at higher contour levels, supports the idea of partial receptor engagement during interaction in the course of capsid transition. We acknowledge that in order to obtain high-resolution cryo-EM reconstruction, the samples were saturated with a large number of TEM8 receptors, a situation that is not valid during a native infection, where there is likely an intricate interplay between the role of pH and the number of receptors bound to the virus.

Overall, this study provides detailed insights into the multiple structural states of the SVV capsid and supports the existence of an SVV A-particle. Future research could explore whether these structural states correspond to specific genome release intermediates during infection *in vivo*. Additionally, our finding that SVV peptide segments may regulate genome release provides insights into the underlying mechanism and opens avenues for optimizing SVV in oncolytic therapy.

## MATERIALS AND METHODS

The experiments were performed as described below. The supplemental material also provides details on data collection, model building, and analysis. Briefly, CryoSPARC version 4.5.3 (58, 59) was used to generate 3D reconstructions. ChimeraX version 1.7.1 (60), Isolde version 1.7.1 (61), Phenix version 1.21.1-5286 (62), and Coot version 0.9.8.8 (63) were used for atomic model refinement. PDBePISA (64) was used for data analysis. PDBsum (65) was used to generate an example figure of the monomer protein topology that comprises the asymmetric unit.

### Virus production and purification

SCLC H446wt cells (ATCC, HTB-171) were cultured in Roswell Park Memorial Institute (RPMI) 1640 medium (catalog no. 1851354; Gibco) supplemented with 10% (vol/vol) fetal bovine serum (FBS) and 1× (vol/vol) penicillin–streptomycin until reaching approximately 80% confluency in eight T175 flasks. To initiate infection, the medium was replaced with RPMI 1640 containing 2% (vol/vol) FBS and plaque-purified SVV stock at a multiplicity of infection of 1, followed by incubation at 37°C for 3 days. After 3 days, the detached cells were subjected to three rounds of freeze-thaw cycles to permeabilize the cell membranes.

The cell suspension was then transferred to 250 mL Nalgene bottles and centrifuged at 10,000 × *g* for 30 minutes at 4°C. The resulting supernatant was collected, distributed into six 38.5 mL open-top polypropylene tubes (catalog no. Z60105SCA; Beckman Coulter), and centrifuged at 120,000 × *g* for 1 hour at 4°C in a Beckman Coulter SW32Ti rotor. After discarding the supernatant, the virus pellet was resuspended in CsCl purification buffer (137 mM NaCl, 5 mM KCl, 25 mM Tris base, and 0.8 mM NaH2PO_4_, pH 7.4) and incubated overnight at 4°C.

The resuspended virus was then layered onto 6 mL of 1.33 g/mL isopycnic CsCl gradient in a 16.5 mL open-top polypropylene tube (catalog no. Z60303SCA; Beckman Coulter) and centrifuged at 96,000 × *g* for 18 hours in a Beckman Coulter SW 32.1 Ti rotor at 22°C. Viral bands were collected and dialyzed overnight at 4°C against PBS buffer. The final virus concentration in the dialyzed sample was determined using a Qubit protein concentration assay kit (catalog no. 1814929; Life Technologies).

### PaSTRy assay

In the PaSTRy assay, SVV was incubated at a viral protein concentration of 1.5 µg in 7.5× SYPRO Orange and PBS, with different receptor ratios at pH 7.4. The receptor ratios tested included 1:600, 1:300, 1:60, and 1:10, along with an SVV-only control. Additionally, a heat-cool-heat sample was prepared by incubating SVV with the receptor at a 1:600 ratio, followed by sequential heating to 37°C, cooling to 4°C, and reheating to 37°C. Samples were loaded into a ViiA 7 Real-Time PCR System (ThermoFisher Scientific) and subjected to a thermal denaturation protocol: a 2 minute hold at 5°C, a temperature ramp from 25°C to 95°C at 0.05°C per second, and a final hold at 95°C for 2 minutes.

### SVV-TEM8 interaction sample preparation

The interaction between SVV and TEM8 was performed following established protocols (46). Briefly, purified SVV capsids (0.25 mg/mL) were combined in equal volumes with recombinant human TEM8-Fc (1 mg/mL) (catalog no. 13367-H02H; Sino Biological), resulting in a ratio of 600 receptor particles per capsid. The virus-receptor mixture was incubated at 37°C for 90 minutes. The control sample was cooled down to 4°C (46), and the other sample was kept at 37°C till the plunge freezing of cryo-EM grids.

### SVV at pH 5 with TEM8 sample preparation

SVV capsid preparation (0.25 mg/mL) was incubated with a mixture of monosodium phosphate and disodium phosphate buffer solution at pH 5.0. The sample was incubated at room temperature for 1 hour. Recombinant human TEM8-Fc (1 mg/mL) (catalog no. 13367-H02H; Sino Biological), resulting in a ratio of 600 receptor particles per capsid, was added to the incubated sample. The virus-receptor mixture was incubated at 37°C for 90 minutes. The sample was kept at 37°C till the plunge freezing of cryo-EM grids.

### Cryo-EM sample preparation and data collection

Cryo-EM samples were prepared by applying 3.5 µL of purified SVV at a concentration of 0.25 mg/mL to glow-discharged Quantifoil R2/1 grids. Samples were blotted for 4.5 seconds at a blot force of 1.0, then rapidly plunged into liquid ethane cooled by liquid nitrogen using a semi-automated FEI Vitrobot MkIV operating at 95% relative humidity and 37°C or 4°C temperature. Vitrified grids were clipped and imaged on an FEI Titan Krios G2 equipped with a bottom-mounted Falcon 3EC detector (Thermo Fisher Scientific) using Thermo Fisher Scientific EPU software. Each image stack was collected with a total dose of 40 electrons/Å² distributed across 40 EPU fractions, with a final calibrated pixel size of 1.4 Å. Frames were motion corrected using MotionCor2, and CTF parameters were estimated using Patch CTF in CryoSPARC version 4.5.3 (58, 59).

### Reconstruction of the A-particle[P]

CryoSPARC version 4.5.3 (58, 59) was employed to generate all reconstructed maps. The A-particle[P] was blob-picked/auto-picked and extracted using a box size of 420 pixels. Good 2D classes were identified by further 2D classification to eliminate junk particles. The optimal class, containing 1,061 particles, underwent several rounds of 2D classification aimed at identifying class diversity. The two best 2D classes, containing 451 and 427 particles, were subjected to *ab initio* reconstruction with C1 symmetry, followed by homogeneous refinement using icosahedral symmetry. The resulting 3D reconstructions were visually inspected in ChimeraX version 1.7.1 (60) and found to be similar.

Subsequently, the best 2D classes were combined and processed through *ab initio* reconstruction with C1 symmetry, then refined via homogenous refinement with icosahedral symmetry. No receptor TEM8-related density was observed in the 3D reconstruction, irrespective of particles extracted with box sizes of 420, 400, 350, or 330 pixels. Among these, the 350-pixel box size reconstruction was selected for homogeneous refinement and non-uniform refinement using icosahedral symmetry.

### Reconstruction of the E^R^-particle[P]

The reconstruction process was carried out using CryoSPARC version 4.5.3 (58, 59). E^R^-particles were manually picked and extracted with a box size of 420 pixels. Multiple rounds of 2D classification were conducted to eliminate junk particles. Junk particles were defined as those with poor resolution of secondary features, those that were obviously non-particles or ice picks. The good 2D classes were selected and used for* ab initio* reconstruction with C1 symmetry, followed by homogeneous refinement with icosahedral symmetry. The density corresponding to the receptor TEM8 showed the most improvement when particles were extracted using a box size of 350 pixels. However, further refinement of the 3D reconstruction using non-uniform refinement did not improve the resolution.

### Reconstruction of the A- and E^R^-particles[C]

The following reconstruction process was performed in CryoSPARC version 4.5.3 (58, 59). The A-particles[C] were blob-picked/auto-picked. The E^R^-particles[C] were manually picked and extracted with a box size of 330 pixels. The good 2D classes were further classified to remove junk particles. The best class was subjected to *ab initio* reconstruction with C1 symmetry and refined by homogeneous refinement and non-uniform refinement with icosahedral symmetry.

### Reconstruction of O-particles under both conditions

The following reconstruction process was performed in CryoSPARC version 4.5.3 (58, 59). The broken particles were manually picked, extracted with a box size of 420 pixels, and subjected to 2D classification. The reconstruction workflow of the broken particles is presented in Fig. S1 and S2.

### Reconstruction of the genome under physiological and acidic conditions

The following reconstruction process was performed in CryoSPARC version 4.5.3 (58, 59). The genomes were blob-picked/auto-picked, extracted with a box size of 300 pixels, and subjected to 2D classification. The best 2D classes were subjected to further 2D classification to remove junk particles. The best 2D classes were subjected to *ab initio* reconstruction with C1 symmetry and refined by homogeneous refinement with C1 and icosahedral symmetry for both samples.

### Atomic model building and refinement

The crystal structure of the asymmetric unit of SVV (PDB: 3CJI) was used as a starting model to build the atomic model of the A and E^R^ particles. The model was manually fitted in the cryo-EM density map using the rigid body fit function (Fit Atomic Model In Map) in ChimeraX version 1.7.1 (60). One asymmetric unit containing VP1, VP2, VP3, and VP4 was selected and segmented, and the surrounding map was excluded to produce a map containing density only for the asymmetric unit. The map was manually refined in Isolde version 1.7.1 (61) within ChimeraX version 1.7.1 (60) and refined in Phenix version 1.21.1-5286 (62) with the phenix.real_space_refine program. Model refinement was performed in Coot version 0.9.8.8 (63) until satisfactory model refinement statistics were obtained in MolProbity (Tables S3 to S6). Interpentamer interactions in the full particle and A-particle were generated from PDBePISA (64). The example figure of protein topology of the monomers that make up the asymmetric unit was generated with PDBsum (65). The model and maps were deposited into the Protein Data Bank server, and the map-model fit calculated by the server is presented in Table S7.

### Comparing A-particle capsid proteins and measuring capsid volume/diameter and genome diameter

The RMSD of all atom pairs was computed by the matchmaker tool in ChimeraX version 1.7.1 (60). The capsid volume was measured using an in-house Python script and ChimeraX version 1.7.1 (60). The diameter of the capsid was measured by measuring the distance between opposing pentamers in the reconstructed capsid. Two pentamers were fitted in the reconstruction so that they are opposing each other. Markers were placed at the center of mass of each pentamer atomic model. The distance between both markers was measured using the distance tool in ChimeraX version 1.7.1 (60). The genome diameter was measured by placing a marker at the center of mass of the map. Two additional markers were placed at a consistent distance up and down around the genome, respectively, from the center of the *y*-axis. The diameter of the genome was measured as a straight-line distance between the two markers placed on opposite ends of the genome. This approach provides an estimate of the genome diameter.

## Data Availability

The maps and models have been deposited in the Protein Data Bank (PDB) and Electron Microscopy Data Bank (EMDB) with accession codes 9EAA and EMD-47827 for A-particle[C], 9EAB and EMD-47828 for A-particle[P], 9EAC and EMD-47829 for E^R^-particle[C], 9EAD and EMD-47830 for E^R^-particle[P].

## SUPPLEMENTAL MATERIAL

Supplemental figures jvi.00744-25-s0001.pdf Figures S1 to S10.

Supplemental tables jvi.00744-25-s0002.pdf Tables S1 to S7.

ASM does not own the copyrights to Supplemental Material that may be linked to, or accessed through, an article. The authors have granted ASM a non-exclusive, world-wide license to publish the Supplemental Material files. Please contact the corresponding author directly for reuse.
