# Supplementary material for: Cryo-EM structure of the Seneca Valley virus A-particle and related structural states
Source: J Virol. 2025 Aug 20;99(9):e00744-25. doi: 10.1128/jvi.00744-25 (PMC12455960; doi:10.1128/jvi.00744-25)
Supplement: Supplemental figures — Figures S1 to S10. [file jvi.00744-25-s0001.pdf]

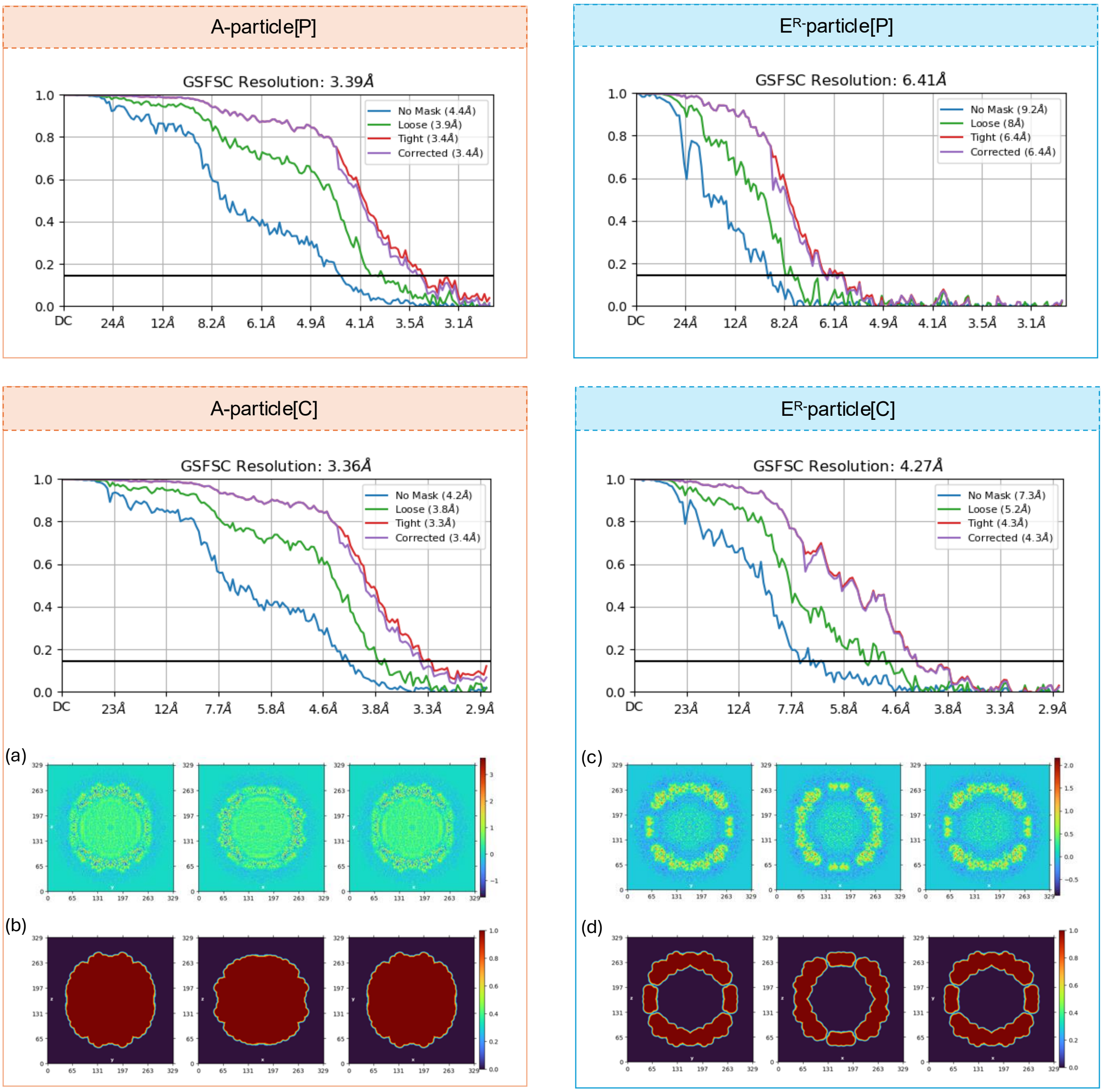

Figure S1: Fourier Shell Correlation Curves and Real-Space Slices for Particle Reconstructions. The Fourier shell correlation (FSC) curves for gold-standard reconstructions of the A-particle[P], A-particle[C], E^R-particle[P], and E^R-particle[C] with icosahedral symmetry are shown. The resolutions for the A-particle[P] and A-particle[C] are 3.39 Å and 3.36 Å, respectively, while the E^R-particle [P] and E^R-particle [C] have resolutions of 6.41 Å and 4.27 Å, respectively. The plots were generated using either homogeneous or non-uniform refinement in CryoSPARC v4.5.3 [51, 52]. (a, c) Three real-space slices through the 3D density along the x, y, and z axes. (b, d) Three real-space slices through the solvent mask along the x, y, and z axes.

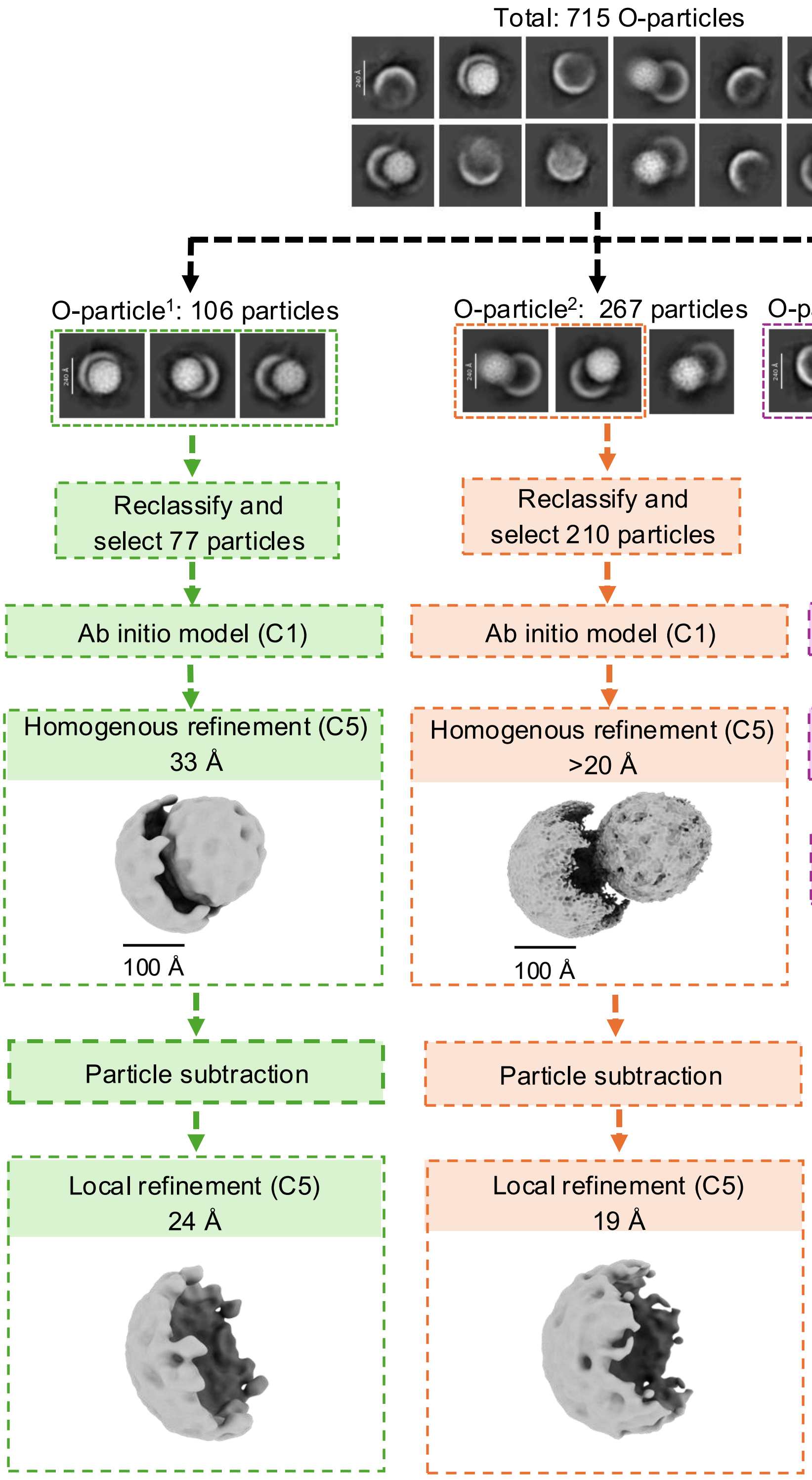

Figure S2: Reconstruction Workflow for SVV Open Particles (O-Particles) at physiological conditions in complex with TEM8. From 554 micrographs, 715 O-particles were manually picked and extracted with a box size of 420px. 2D classification grouped the particles into O-particle[1] (green), O-particle[2] (orange), and O-particle[3]/[4] (purple). For O-particle[1] and O-particle[2], refinement achieved capsid resolutions of 24 Å and 19 Å, respectively. Subtraction of genomic density improved resolution, but refinement without subtraction was unsuccessful. For O-particle[3] and O-particle[4], re-classification and ab initio modeling did not yield high-quality reconstructions, even when using an A-particle as a reference. Non-uniform refinement did not improve resolution for any particle group.

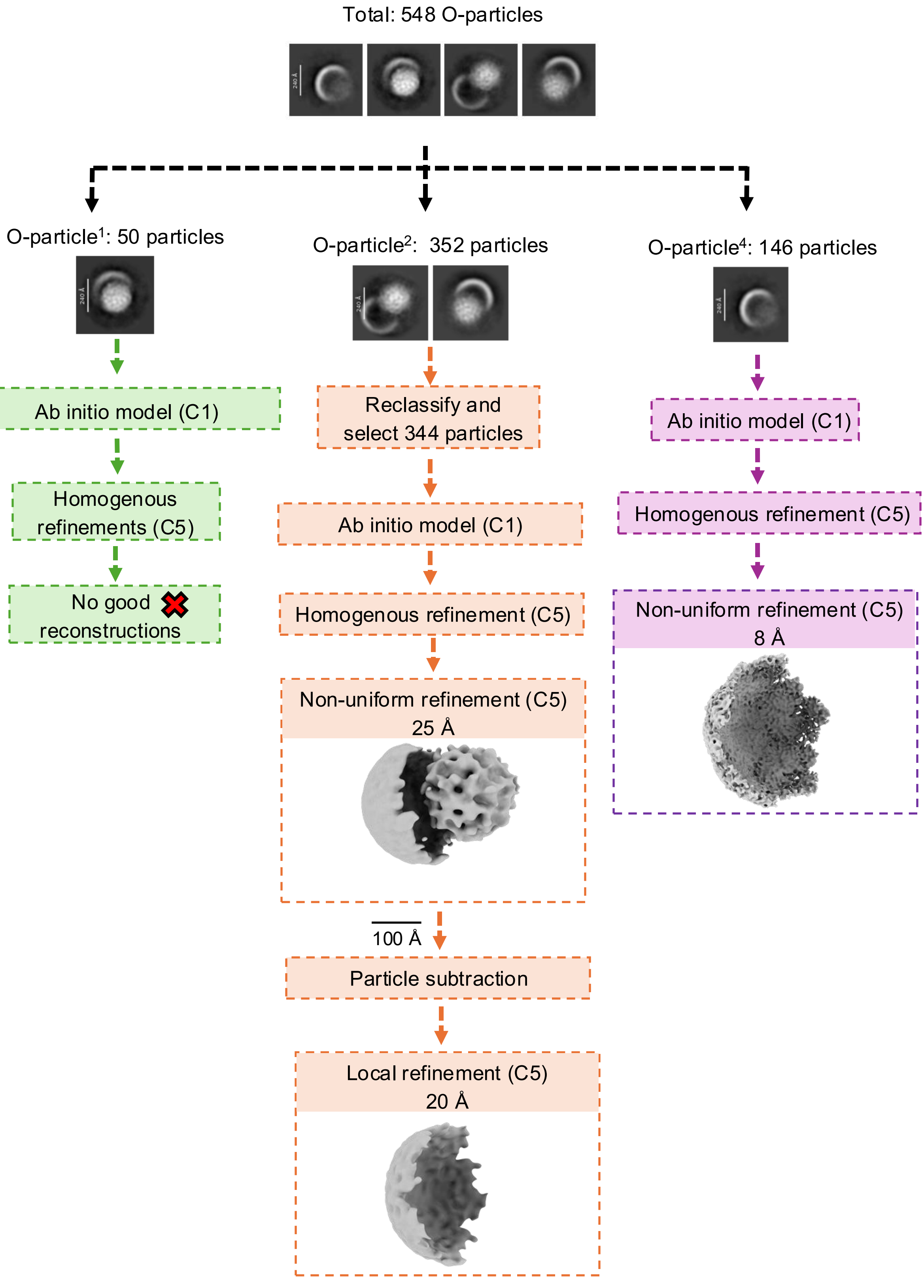

Figure S3: Reconstruction Workflow for SVV Open Particles (O-Particles) at Acidic Conditions. Reconstruction of SVV Open particles (O-particles) formed under acidic conditions is shown. From 682 micrographs, 548 O-particles were manually picked and extracted with a box size of 420px. 2D classification grouped the particles into O-particle[1] (green), O-particle[2] (orange), and O-particle[4] (purple). O-particle[1]: 50 particles were refined but did not yield high-quality reconstructions. O-particle[2]: 344 particles were used for reconstruction after genome subtraction, achieving a capsid resolution of 19.58 Å. O-particle[4]: 146 particles were refined, resulting in a capsid resolution of 7.83 Å. The workflow included 2D classification, ab initio model generation, homogeneous refinement, non-uniform refinement, and localized refinement

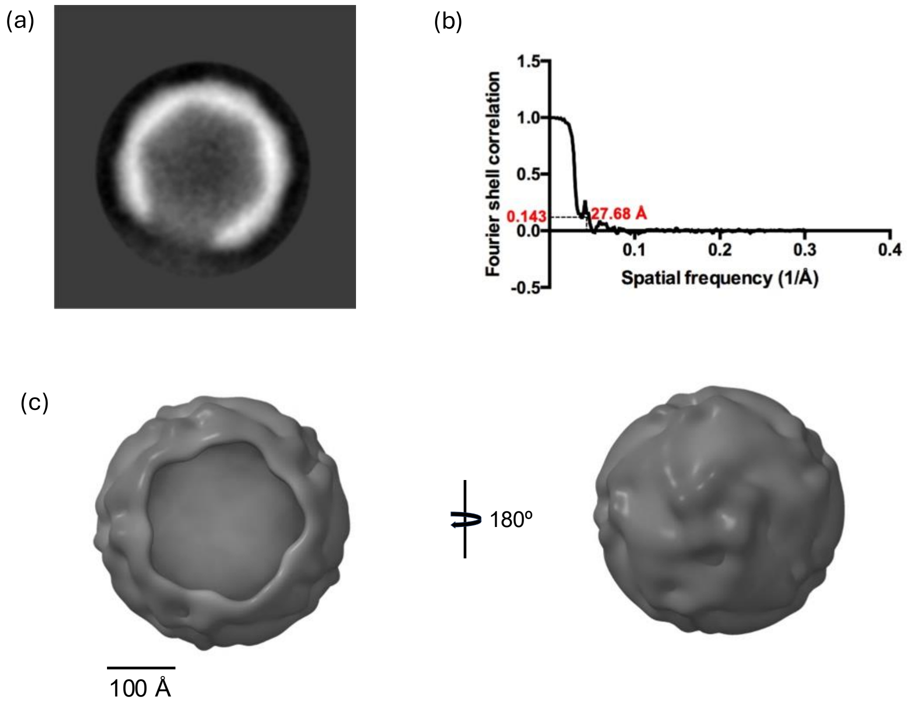

Figure S4: Reconstruction of SVV empty capsids with one pentamer missing. SVV F-particle and procapsids are naturally occurring at pH7. **(a)** 2D classification of SVV procapsid particles, revealed a class average of capsids with one pentamer missing (O-particle). **(b)** The Fourier shell correlation curve for the gold standard reconstruction of the O-particle. **(c)** Radially colored SVV-1p map viewed down the 5-fold axis rotated 180º clockwise. (Scale bar 100 Å)

(a)

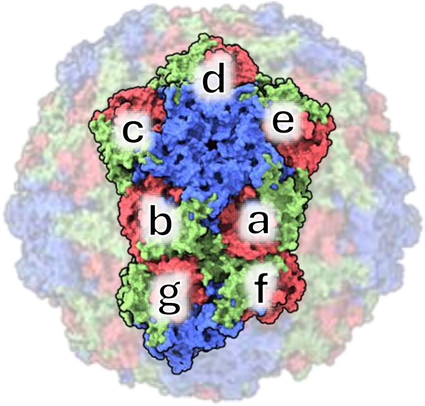

(b)

Interface		Buried surface area (Å2)		
		F-particle	A-particle[P]	A-particle[C]
Intra-protomer	VP1a-VP3a	2994	2854	2936
	VP1a-VP2a	2618	2511	2582
	VP1a-VP4a	478	435	417
	VP2a-VP3a	1590	1406	1423
	VP3a-VP4a	776	761	809
	VP2a-VP4a	355	333	295
	**Sum**	**8812**	**8300**	**8462**
	**Percentage change**		**-6%**	**-4%**

(c)

Interface		Buried surface area (Å2)		
		F-particle	A-particle[P]	A-particle[C]
Inter-pentamer	VP3b-VP3g	99	38	58
	VP3b-VP2g	13	-	-
	VP2b-VP3g	1465	1093	1257
	VP2b-VP2f	751	484	544
	VP2b-VP1g	243	103	165
	VP2b-VP2g	53	-	-
	VP1a-VP2f	241	103	165
	VP3a-VP2f	1468	1098	1258
	VP3a-VP3f	99	39	58
	VP2a-VP2f	53	-	-
	VP2a-VP3f	13	-	-
	**Sum**	**4498**	**2958**	**3504**
	**Percentage change**		**-34%**	**-22%**

(d)

Interface		Buried surface area (Å2)		
		F-particle	A-particle[P]	A-particle[C]
Intra-pentamer	VP1a-VP3e	1407	1269	1286
	VP1a-VP1b	1060	857	867
	VP1a-VP1e	1057	864	856
	VP1a-VP2b	190	115	135
	VP1a-VP3b	174	142	166
	VP1a-VP4b	125	109	86
	VP1a-VP3d	5	-	-
	VP3a-VP1b	1411	1264	1291
	VP3a-VP2b	852	760	788
	VP3a-VP3e	759	764	748
	VP3a-VP3b	758	762	751
	VP3a-VP1e	174	142	165
	VP3a-VP3d	166	123	138
	VP3a-VP3c	165	124	137
	VP3a-VP4b	161	121	148
	VP3a-VP4d	151	144	145
	VP3a-VP4e	55	22	18
	VP3a:[CA]e	13	12	11
	VP3a:[CA]d	10	13	11
	VP3a:[CA]b	8	12	11
	VP3a:[CA]c	7	12	11
	[CA]a: VP3b	13	12	10
	[CA]a: VP3a	12	11	10
	[CA]a: VP3c	10	13	11
	[CA]a: VP3e	8	11	12
	[CA]a:VP3d	7	12	12
	[CA]a:[CA]b	13	-	-
	[CA]a:[CA]e	13	-	-
	[CA]a:[CA]d	12	-	-
	[CA]a:[CA]c	12	-	-
	VP3a-VP4c	6	-	-
	VP3a-VP1c	5	-	-
	VP2a-VP3e	858	760	782
	VP2a-VP1e	191	116	133
	VP4a-VP4e	265	225	215
	VP4a-VP4b	265	224	216
	VP4a-VP3e	161	121	149
	VP4a-VP3c	149	144	146
	VP4a-VP1e	123	110	85
	VP4a-VP3b	54	22	18
	VP4a-VP3d	6	-	-
	**Sum**	**10891**	**9408**	**9566**
	**Percentage change**		**-14%**	**-12%**

Figure S5: Buried Surface Area (Å²) of Interfaces within the SVV Full Particle (F-particle), A-particle[P], and A-particle[C]. The buried surface area at different interfaces within the SVV particles. (a) Capsid surface representation of the full particle, with VP1, VP2, and VP3 subunits shown in blue, green, and red, respectively. The VP4 subunit is located on the interior of the capsid and is colored yellow. The units used for buried surface area calculation are labeled from a to g. (b) Intra-protomer, (c) Inter-pentamer, and (d) Intra-pentamer buried surface areas were calculated using the PDBePISA server [57]. These models were built in their respective maps, with minor differences in side-chain densities. Some side chains were removed during modeling, this potentially results in the observed differences between the A-particles

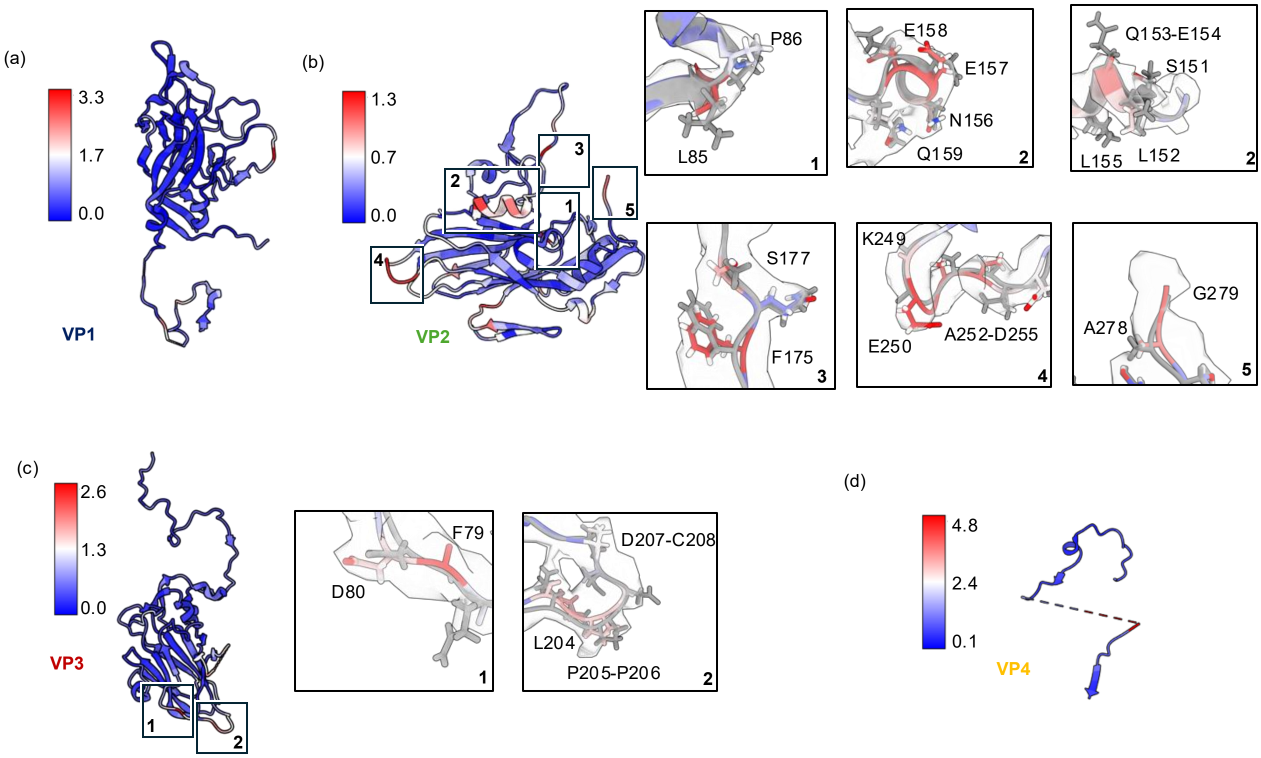

Figure S6: Comparison of A-particle Atomic Models. The structural differences between the A-particle[P] and A-particle[C] were analyzed by calculating shifts in Cα positions using ChimeraX v1.7.1 [57]. The atomic model of A-particle[C] is shown in grey, with RMSD of all atom pairs mapped onto the A-particle[P], where blue represents minimal changes and red indicates the largest changes. The RMSD values for VP1, VP2, VP3, and VP4 are 0.563 Å, 0.465 Å, 0.522 Å, and 0.980 Å, respectively, with lower RMSD values indicating greater similarity. (a) VP1 shows minimal differences in the N terminus. (b) VP2 shows shifts in labelled residues not exceeding 1.3 Å. (c) VP3 shows shifts of up to 2.6 Å in labelled residues. (d) VP4 shows minimal differences, except in partially ordered regions.

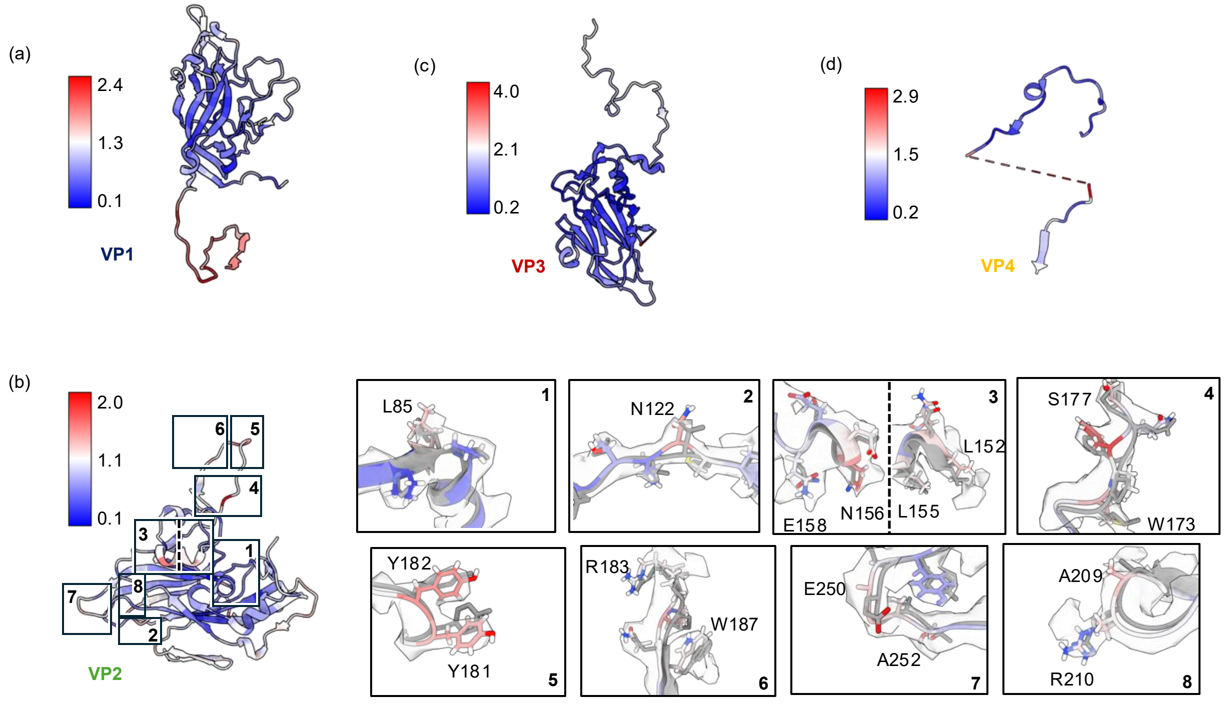

Figure S7: Structural Changes in the Capsid Proteins at Acidic Conditions. The structural changes in the capsid proteins of the A-particle[C] relative to the SVV F-particle crystal structure (PDB ID: 3CJI) were analyzed by calculating shifts in Cα positions using ChimeraX v1.7.1 [53]. The SVV crystal structure (PDB ID: 3CJI) is shown in grey, and the RMSD of all atom pairs between the two structures are mapped onto the A-particle[C], where blue represents minimal changes and red indicates the largest changes. The RMSD values for VP1, VP2, VP3, and VP4 are 0.976 Å, 0.840 Å, 1.067 Å, and 0.945 Å, respectively, with lower RMSD values indicating higher structural similarity. (a) VP1 shows minimal differences in the partially ordered N terminus. (b) VP2 shows shifts in labelled residues not exceeding 2.0 Å. (c) VP3 shows minimal differences. (d) VP4 shows minimal differences, except in partially ordered regions.

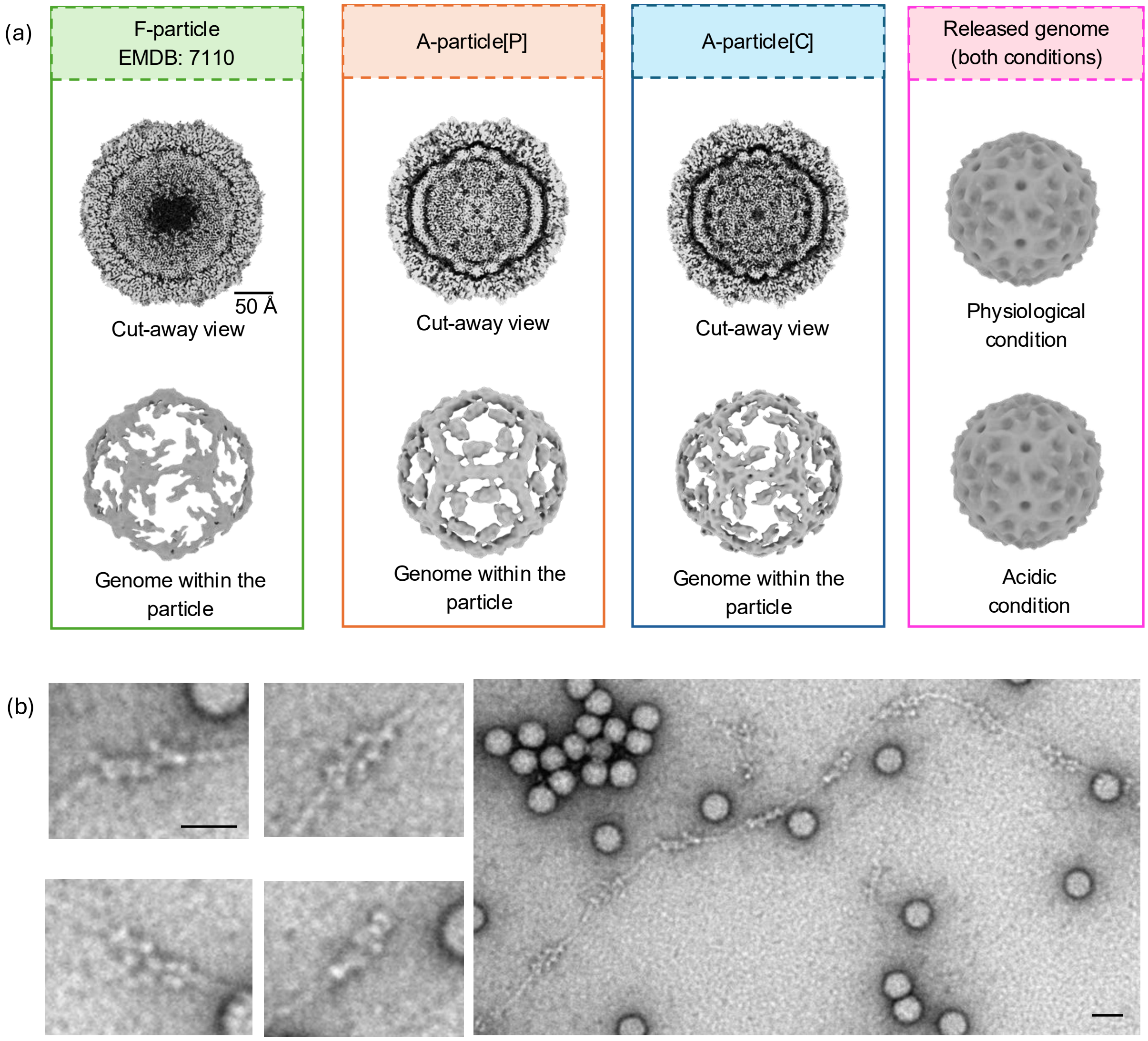

Figure S8: Electron Density of the Genome in the SVV F-particle, A-particle[P], A-particle[C], and Isolated Genome. The particles were reconstructed with Icosahedral symmetry. In the top row, the genome in the F-particle is uniformly distributed near the capsid. In the A-particle[P] and A-particle[C], genome is centrally located with no contact to the capsid. In the bottom row, after excluding the capsid and filtering the genome densities to 10 Å, the genome region closer to the capsid is ordered into a dodecahedral cage. However, the released genome, reconstructed with Icosahedral symmetry, does not display the dodecahedral cage (scale bar 50 Å). Additionally, panel (b) shows occasional behavior of the SVV capsid at low pH, where extended RNA strings alternate with unfolded regions, as observed in negative staining (scale bar 300 Å).

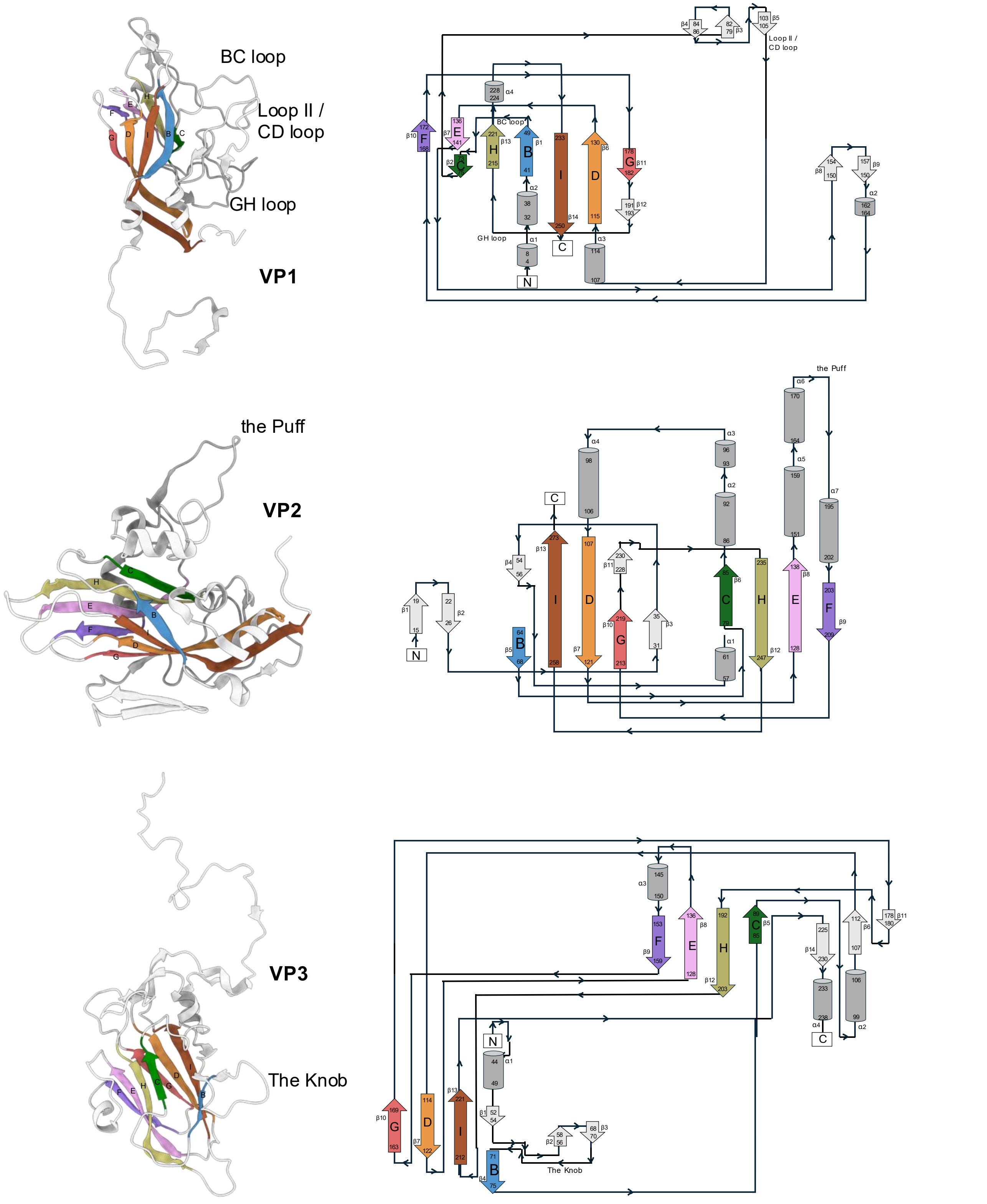

Figure S9: Secondary structure topology of the major capsid proteins VP1, VP2 and VP3 of Seneca Valley virus (PDB-ID: 3cji). Ribbon diagram of the major capsid proteins (left) and schematic topology diagram (right). The β-sheets are represented as arrows and α-helices as cylinders. The values within the arrows and cylinder represents the amino acid sequence that makes the secondary structures. The β-sheets that make up the single jellyroll is represented as B-I in various colors. The other sheets and helices are labelled β(sequence) and α(sequence).

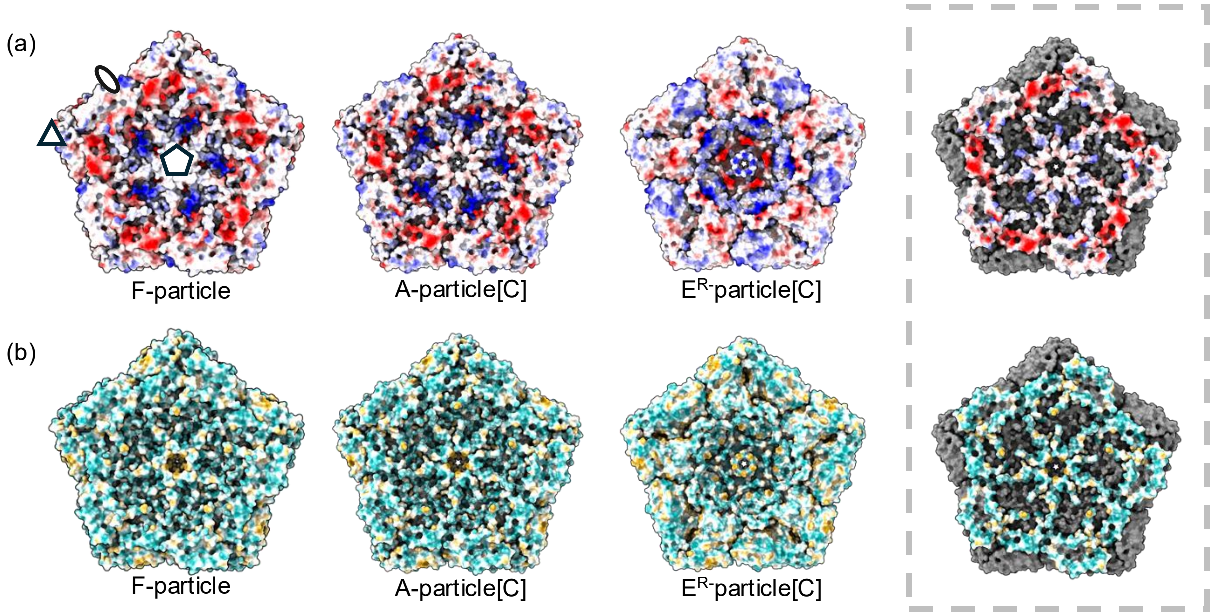

Figure S10: Comparison of Electrostatic and Hydrophobicity Interior Capsid Surface (a) Electrostatic surface comparison of the SVV F-particle (PDB: 3CJI), A-particle[C], E^R-particle[C], and regions lacking density in the E^R-particle[C] (grey box). The five-, three-, and two-fold symmetry axes are marked with a pentagon, triangle, and oval, respectively, The color scale ranges from red (negative) to blue (positive), with values from -10 to 10 kT/e. (b) Hydrophobicity surface comparison, with blue indicating the most hydrophilic areas and yellow indicating the most hydrophobic. The color scale ranges from -20 to 20. Surface calculations were performed using ChimeraX version 1.7.1 [53].
