# Supplementary material for: Cryo-EM structure of the Seneca Valley virus A-particle and related structural states
Source: J Virol. 2025 Aug 20;99(9):e00744-25. doi: 10.1128/jvi.00744-25 (PMC12455960; doi:10.1128/jvi.00744-25)
Supplement: Supplemental tables — Tables S1 to S7. [file jvi.00744-25-s0002.pdf]

Supporting Information for

Cryo-EM Structure of the Seneca Valley virus A-Particle and Related Structural States

Rosheny Kumaran[1], Kuan-Lin Chen[2], Nadishka Jayawardena[1,2], Alice-Roza Eruera[1], James Hodgkinson-Bean[1], Laura Burga[1], Matthias Wolf[2], Mihnea Bostina[1*]

[1]Department of Microbiology and Immunology, University of Otago, Dunedin, New Zealand

[2]Molecular Cryo-Electron Microscopy Unit, Okinawa Institute of Science and Technology Graduate University, Okinawa, Japan

***Corresponding author:** Mihnea Bostina, University of Otago, 720 Cumberland Street, Dunedin, 9016, Otago, New Zealand

Corresponding author: Mihnea Bostina
Email: mihnea.bostina@otago.ac.nz

This PDF file includes:

Tables S1 to S7
Figures S1 to S10

Tables S1 to S7

Table S1: Cryo-EM and Reconstruction information. Data collection parameters, including reconstruction and refinement details

**Data collection**				
Microscope	FEI Titan Krios G2	FEI Titan Krios G2	FEI Titan Krios G2	FEI Titan Krios G2
Detector	Falcon 3EC	Falcon 3EC	Falcon 3EC	Falcon 3EC
Pixel size (Å)	1.4	1.4	1.4	1.4
Voltage (kV)	300	300	300	300
Electron dose (e-/Å²)	40	40	40	40
**Reconstruction**	**A-particle[P]**	**E^R-particle[P]**	**A-particle[C]**	**E^R-particle[C]**
Software	CryoSPARC v4.5.3 [1, 2]	CryoSPARC v4.5.3 [1, 2]	CryoSPARC v4.5.3 [1, 2]	CryoSPARC v4.5.3 [1, 2]
Number of micrographs	706	706	855	855
Extraction box size (pix)	350	350	330	330
Number of particles in the reconstruction	858	137	817	242
Symmetry	I	I	I	I
Fourier shell correlation (FSC) cut-off	0.143	0.143	0.143	0.143
Final resolution	3.39 Å	6.41 Å	3.36 Å	4.27 Å
**Atomic model refinement**				
Softwares	Isolde v1.7.1 [3], Phenix v1.21.1-5286 [4] and Coot v0.9.8.8 [5]			

Table S2: Volume and Diameter Comparison of the Viral Particles. Volume (Å³) and diameter (Å) measurements are provided for the Full particle, Procapsid, A-particle[P], A-particle[C], E^R-particle[P], and E^R-particle[C]. Percentage increases in volume/diameter are shown for the A-particle and E^R-particle relative to the F-particle. The diameter of expelled genomes is compared to the internal genome of the A-particle, with the change expressed as a percentage. All values were calculated in ChimeraX v1.7.1 [6]. Additional buried surface analysis were done on these particles using the PDBePISA server [7] and presented as Figure S5.

Measurements (unit)	Particle type	Values	Difference
**Volume (Å³)**	F-particle	9.10×10^6 Å³	
	Procapsid	9.28×10^6 Å³	2% increase
	A-particle[P]	1.07×10^7 Å³	18% increase
	A-particle[C]	1.06×10^7 Å³	16% increase
	E^{R-}particle[P]	1.32×10^7 Å³	45% increase
	E^{R-}particle[C]	1.31×10^7 Å³	44% increase
**Diameter (Å)**	F-particle	243 Å	
	Procapsid	244 Å	<1% increase
	A-particle[P]	257 Å	6% increase
	A-particle[C]	256 Å	5% increase
	E^{R-}particle[P]	278 Å	14% increase
	E^{R-}particle[C]	277 Å	14% increase
	A-particle[P] internal genome	220 Å	
	A-particle[C] internal genome	218 Å	
	Expelled genome[P]	228 Å	4% increase
	Expelled genome[C]	220 Å	1% increase

Table S3 and S4: Validation of Capsid Asymmetric Unit Models. Validation tables for the deposited capsid asymmetric unit model of the A-particle[P] and the A-particle[C]. Phenix v1.21.1-5286 [4].

**Model: A-particle[P]**	
**Refinement map resolution: 3.39 Å**	
**Composition (#)**	
Chains	5
Atoms	11733 (Hydrogens: 5700)
Residues	Protein: 789 Nucleotide: 0
Water	0
Ligands	CA: 1
**Bonds (RMSD)**	
Length (Å) (# > 4σ)	0.003 (0)
Angles (°) (# > 4σ)	0.518 (0)
**MolProbity score**	**1.55**
**Clash score**	**3.17**
**Ramachandran plot (%)**	
Outliers	0.13
Allowed	6.61
Favored	93.26
**Rama-Z (Ramachandran plot Z-score RMSD)**	
whole (N = 771)	-1.11 (0.30)
helix (N = 67)	-0.54 (0.64)
sheet (N = 160)	-0.27 (0.41)
loop (N = 544)	-0.93 (0.27)
**Rotamer outliers (%)**	**0.63**
**Cβ outliers (%)**	**NA**
**Peptide plane (%)**	
Cis proline/general	3.0/0.0
Twisted proline/general	0.0/0.0
**CaBLAM outliers (%)**	**2.26**
**ADP (B-factors)**	
**Iso/Aniso (#)**	**6033/0**
**min/max/mean**	
Protein	70.20/212.34/110.35
Nucleotide	---
Ligand	70.45/70.45/70.45
Water	---
**Occupancy**	
**Mean**	**1**
**occ = 1 (%)**	**100**
**0 < occ < 1 (%)**	**0**
**occ > 1 (%)**	**0**
**Model vs. Data**	
CC (mask)	0.82
CC (box)	0.85
CC (peaks)	0.86
CC (volume)	0.83
Mean CC for ligands	0.95

**Model: A-particle[C]**	
**Refinement map resolution: 3.36 Å**	
**Composition (#)**	
Chains	5
Atoms	11927 (Hydrogens: 5826)
Residues	Protein: 791 Nucleotide: 0
Water	0
Ligands	CA: 1
**Bonds (RMSD)**	
Length (Å) (# > 4σ)	0.005 (0)
Angles (°) (# > 4σ)	0.606 (6)
**MolProbity score**	**1.5**
**Clash score**	**3.2**
**Ramachandran plot (%)**	
Outliers	0
Allowed	5.65
Favored	94.35
**Rama-Z (Ramachandran plot Z-score RMSD)**	
whole (N = 779)	-1.94 (0.28)
helix (N = 68)	-1.91 (0.59)
sheet (N = 191)	-0.84 (0.37)
loop (N = 520)	-1.42 (0.25)
**Rotamer outliers (%)**	**0**
**Cβ outliers (%)**	**NA**
**Peptide plane (%)**	
Cis proline/general	0.0/0.0
Twisted proline/general	0.0/0.0
**CaBLAM outliers (%)**	**2.35**
**ADP (B-factors)**	
**Iso/Aniso (#)**	**6101/0**
**min/max/mean**	
Protein	49.41/174.14/89.27
Nucleotide	---
Ligand	40.43/40.43/40.43
Water	---
**Occupancy**	
**Mean**	**1**
**occ = 1 (%)**	**100**
**0 < occ < 1 (%)**	**0**
**occ > 1 (%)**	**0**
**Model vs. Data**	
CC (mask)	0.84
CC (box)	0.83
CC (peaks)	0.83
CC (volume)	0.84
Mean CC for ligands	0.94

Table S5 and S6: Validation of Capsid Asymmetric Unit Models. Validation tables for the deposited capsid asymmetric unit model of the E^R-particle[P] and the E^R-particle[C]. Tables were generated using Phenix v1.21.1-5286 [4].

**Model: ER-particle[P]**	
**Refinement map resolution: 6.41 Å**	
**Composition (#)**	
Chains	4
Atoms	4103 (Hydrogens: 1354)
Residues	Protein: 687 Nucleotide: 0
Water	0
Ligands	CA: 1
**Bonds (RMSD)**	
Length (Å) (# > 4σ)	0.002 (0)
Angles (°) (# > 4σ)	0.718 (0)
**MolProbity score**	1.73
**Clash score**	4.63
**Ramachandran plot (%)**	
Outliers	0.15
Allowed	7.93
Favored	91.92
**Rama-Z (Ramachandran plot Z-score RMSD)**	
whole (N = 681)	-2.75 (0.29)
helix (N = 50)	-2.07 (0.60)
sheet (N = 156)	-1.65 (0.38)
loop (N = 475)	-1.97 (0.27)
**Rotamer outliers (%)**	0
**Cβ outliers (%)**	NA
**Peptide plane (%)**	
Cis proline/general	3.2/0.0
Twisted proline/general	0.0/0.0
**CaBLAM outliers (%)**	**4.44**
**ADP (B-factors)**	
**Iso/Aniso (#)**	**2749/0**
**min/max/mean**	
Protein	202.42/503.51/278.37
Nucleotide	---
Ligand	40.47/40.47/40.47
Water	---
**Occupancy**	
**Mean**	**1**
**occ = 1 (%)**	**99.98**
**0 < occ < 1 (%)**	**0.02**
**occ > 1 (%)**	**0**
**Model vs. Data**	
CC (mask)	0.69
CC (box)	0.89
CC (peaks)	0.55
CC (volume)	0.65
Mean CC for ligands	0.4

**Model: ER-particle[C]**	
**Refinement map resolution: 4.27 Å**	
**Composition (#)**	
Chains	4
Atoms	8625 (Hydrogens: 3863)
Residues	Protein: 679 Nucleotide: 0
Water	0
Ligands	CA: 1
**Bonds (RMSD)**	
Length (Å) (# > 4σ)	0.002 (0)
Angles (°) (# > 4σ)	0.555 (0)
**MolProbity score**	1.61
**Clash score**	5.02
**Ramachandran plot (%)**	
Outliers	0
Allowed	4.92
Favored	95.08
**Rama-Z (Ramachandran plot Z-score RMSD)**	
whole (N = 671)	-1.84 (0.30)
helix (N = 66)	-1.53 (0.58)
sheet (N = 124)	-0.37 (0.44)
loop (N = 481)	-1.50 (0.27)
**Rotamer outliers (%)**	0
**Cβ outliers (%)**	NA
**Peptide plane (%)**	
Cis proline/general	3.3/0.0
Twisted proline/general	0.0/0.0
**CaBLAM outliers (%)**	**2.56**
**ADP (B-factors)**	
**Iso/Aniso (#)**	**4762/0**
**min/max/mean**	
Protein	114.87/256.72/154.85
Nucleotide	---
Ligand	151.37/151.37/151.37
Water	---
**Occupancy**	
**Mean**	**1**
**occ = 1 (%)**	**100**
**0 < occ < 1 (%)**	**0**
**occ > 1 (%)**	**0**
**Model vs. Data**	
CC (mask)	0.8
CC (box)	0.91
CC (peaks)	0.73
CC (volume)	0.8
Mean CC for ligands	0.83

Table S7: Map-model fit summary. Values were calculated by the Protein Database Deposition server [8] when data are deposited to the PDB. The average atom inclusion of the A-particle [P], A-particle [C], E^R-particle [P] and E^R-particle [C] at the recommended contour level of 0.801, 0.801, 0.6 and 0.726, respectively. The Q-score for the entire model and chain was calculated.

	Chain	Atom inclusion	Q-score
**A-particle [P]**	All	0.8440	0.5110
	A	0.8380	0.5210
	B	0.8590	0.5070
	C	0.8500	0.5070
	D	0.6360	0.4970
**A-particle [C]**	All	0.8260	0.5300
	A	0.8230	0.5300
	B	0.8560	0.5310
	C	0.8490	0.5290
	D	0.6790	0.5330
**E^R-particle [P]**	All	0.9690	0.3080
	A	0.9740	0.3270
	B	0.9470	0.3050
	C	0.9790	0.2920
**E^R-particle [C]**	All	0.8400	0.3960
	A	0.8320	0.4170
	B	0.8410	0.3990
	C	0.8370	0.3700

Supplementary references

1. Punjani A, Rubinstein JL, Fleet DJ, Brubaker MA. CryoSPARC: algorithms for rapid unsupervised cryo-EM structure determination. Nature Methods. 2017;14(3):290-6. doi: 10.1038/nmeth.4169. PubMed PMID: 28165473.

2. Punjani A, Zhang H, Fleet DJ. Non-uniform refinement: adaptive regularization improves single-particle cryo-EM reconstruction. Nature Methods. 2020;17(12):1214-21. doi: 10.1038/s41592-020-00990-8. PubMed PMID: 33257830.

3. Croll TI. ISOLDE: A physically realistic environment for model building into low-resolution electron-density maps. Acta Crystallographica Section D, Structural Biology. 2018;74(Pt 6):519-30. Epub 20180411. doi: 10.1107/s2059798318002425. PubMed PMID: 29872003; PubMed Central PMCID: PMC6096486.

4. Liebschner D, Afonine PV, Baker ML, Bunkóczi G, Chen VB, Croll TI, et al. Macromolecular structure determination using X-rays, neutrons and electrons: recent developments in Phenix. Acta Crystallographica Section D, Structural Biology. 2019;75(Pt 10):861-77. Epub 20191002. doi: 10.1107/s2059798319011471. PubMed PMID: 31588918; PubMed Central PMCID: PMC6778852.

5. Emsley P, Lohkamp B, Scott WG, Cowtan K. Features and development of Coot. Acta Crystallogr D Biol Crystallogr. 2010;66(Pt 4):486-501. Epub 20100324. doi: 10.1107/s0907444910007493. PubMed PMID: 20383002; PubMed Central PMCID: PMC2852313.

6. Pettersen EF, Goddard TD, Huang CC, Meng EC, Couch GS, Croll TI, et al. UCSF ChimeraX: structure visualization for researchers, educators, and developers. Protein Science : a publication of

the Protein Society. 2021;30(1):70-82. Epub 20201022. doi: 10.1002/pro.3943. PubMed PMID: 32881101; PubMed Central PMCID: PMC7737788.
7. Krissinel E, Henrick K. Inference of macromolecular assemblies from crystalline state. Journal of Molecular Biology. 2007;372(3):774-97. doi: 10.1016/j.jmb.2007.05.022.
8. consortium w. Protein Data Bank: the single global archive for 3D macromolecular structure data. Nucleic Acids Research. 2018;47(D1):D520-D8. doi: 10.1093/nar/gky949.
9. Butan C, Filman DJ, Hogle JM. Cryo-electron microscopy reconstruction shows Poliovirus 135S particles poised for membrane interaction and RNA release. Journal of Virology. 2014;88(3):1758-70. doi: doi:10.1128/jvi.01949-13. PubMed PMID: 24257617; PubMed Central PMCID: PMC3911577.
10. Shah PNM, Filman DJ, Karunatilaka KS, Hesketh EL, Groppelli E, Strauss M, et al. Cryo-EM structures reveal two distinct conformational states in a Picornavirus cell entry intermediate. PLoS Pathogens. 2020;16(9):e1008920. Epub 20200930. doi: 10.1371/journal.ppat.1008920. PubMed PMID: 32997730; PubMed Central PMCID: PMC7549760.
11. Belnap David M, Filman David J, Trus Benes L, Cheng N, Booy Frank P, Conway James F, et al. Molecular tectonic model of virus structural transitions: The putative cell entry states of Poliovirus. Journal of Virology. 2000;74(3):1342-54. doi: 10.1128/jvi.74.3.1342-1354.2000.
12. Hogle JM, Chow M, Filman DJ. Three-dimensional structure of poliovirus at 2.9 A resolution. Science. 1985;229(4720):1358-65. doi: 10.1126/science.2994218. PubMed PMID: 2994218.
13. Basavappa R, Syed R, Flore O, Icenogle JP, Filman DJ, Hogle JM. Role and mechanism of the maturation cleavage of VP0 in poliovirus assembly: structure of the empty capsid assembly intermediate at 2.9 A resolution. Protein Sci. 1994;3(10):1651-69. doi: 10.1002/pro.5560031005. PubMed PMID: 7849583; PubMed Central PMCID: PMC2142606.
14. Xu L, Zheng Q, Li S, He M, Wu Y, Li Y, et al. Atomic structures of Coxsackievirus A6 and its complex with a neutralizing antibody. Nature Communications. 2017;8(1):505. doi: 10.1038/s41467-017-00477-9.
15. Büttner CR, Spurný R, Füzik T, Plevka P. Cryo-electron microscopy and image classification reveal the existence and structure of the coxsackievirus A6 virion. Communications Biology. 2022;5(1):898. doi: 10.1038/s42003-022-03863-2.
16. Shingler KL, Yoder JL, Carnegie MS, Ashley RE, Makhov AM, Conway JF, et al. The Enterovirus 71 A-particle forms a gateway to allow genome release: a CryoEM study of Picornavirus uncoating. PLOS Pathogens. 2013;9(3):e1003240. doi: 10.1371/journal.ppat.1003240. PubMed PMID: 23555253; PubMed Central PMCID: PMC3605244.
17. Wang X, Peng W, Ren J, Hu Z, Xu J, Lou Z, et al. A sensor-adaptor mechanism for enterovirus uncoating from structures of EV71. Nature Structural & Molecular Biology. 2012;19(4):424-9. doi: 10.1038/nsmb.2255. PubMed PMID: 22388738; PubMed Central PMCID: PMC3378640.
18. Venkataraman S, Reddy SP, Loo J, Idamakanti N, Hallenbeck PL, Reddy VS. Structure of Seneca Valley Virus-001: an oncolytic picornavirus representing a new genus. Structure. 2008;16(10):1555-61. doi: 10.1016/j.str.2008.07.013. PubMed PMID: 18940610; PubMed Central PMCID: PMC2572565.
19. Strauss M, Jayawardena N, Sun E, Easingwood RA, Burga LN, Bostina M. Cryo-Electron microscopy structure of Seneca Valley Virus procapsid. Journal of Virology. 2018;92(6). Epub 20180226. doi: 10.1128/jvi.01927-17. PubMed PMID: 29263256; PubMed Central PMCID: PMC5827394.
20. Mullapudi E, Nováček J, Pálková L, Kulich P, Lindberg AM, Kuppeveld FJMv, et al. Structure and genome release mechanism of the human Cardiovirus Saffold Virus 3. Journal of Virology. 2016;90(17):7628-39. doi: doi:10.1128/jvi.00746-16. PubMed PMID: 27279624; PubMed Central PMCID: PMC4988150.
21. Malik N, Kotecha A, Gold S, Asfor A, Ren J, Huiskonen JT, et al. Structures of foot and mouth disease virus pentamers: Insight into capsid dissociation and unexpected pentamer reassociation. PLOS Pathogens. 2017;13(9):e1006607. doi: 10.1371/journal.ppat.1006607. PubMed PMID: 28937999; PubMed Central PMCID: PMC5656323.
22. Fry EE, Newman JWI, Curry S, Najjam S, Jackson T, Blakemore W, et al. Structure of Foot-and-mouth disease virus serotype A10 61 alone and complexed with oligosaccharide receptor: receptor conservation in the face of antigenic variation. J Gen Virol. 2005;86(Pt 7):1909-20. doi: 10.1099/vir.0.80730-0. PubMed PMID: 15958669.
